# Supplementary material for: The UHPLC-Orbitrap MS/MS and Network Pharmacology Strategies Reveal the Active Antioxidants of Bleeding Sap from Sponge Gourd in Treating Tuberculosis
Source: Int J Mol Sci. 2025 Oct 21;26(20):10231. doi: 10.3390/ijms262010231 (PMC12565323; doi:10.3390/ijms262010231)
Supplement: Supplementary file 1 [file ijms-26-10231-s001.zip › Support information File 1 Compounds Identification.pdf]

The UHPLC-Orbitrap MS/MS and Network Pharmacology Strategies Reveal the Active Anti-Oxidants of Bleeding Sap from Sponge Gourd in Treating Tuberculosis

Di Zhang [1], Lu Jiang [1], Tuanjie Wang [3], Yujiang Dai [1], Xinxin Si [1], Huifang Li [1,*], Komal Anjum [2,*]

1 Jiangsu Institute of Marine Resources Development, Jiangsu Ocean University, Lianyungang, 222005, PR China;
2 Department of Medicine and Pharmacy, Ocean University of China, Qingdao, 222005, PR China;
3 Jiangsu Key Laboratory on Innovation for Marine Medicine and Modern Chinese Medicine, Jiangsu Kanion Pharmaceutical Co. Ltd., Lianyungang, 222005, PR China;

*Corresponding authors: huifangli@jou.edu.cn; komalazam@ymail.com

Content

Figure S1. The subfraction of the n-butanol extraction P2 from bleeding sap 1

Figure S2. The MS^1 spectrum of nonanoic acid (**1**) 1

Figure S3. The MS^2 spectrum of nonanoic acid (**1**) 2

Figure S4. The MS^1 spectrum of 3-hydroxy myristic acid (**2**) 2

Figure S5. The MS^2 spectrum of 3-hydroxy myristic acid (**2**) 3

Figure S6. The MS^1 spectrum of decanoic acid (**3**) 3

Figure S7. The MS^2 spectrum of decanoic acid (**3**) 4

Figure S8. The MS^1 spectrum of tridecylic acid (**4**) 4

Figure S9. The MS^2 spectrum of tridecylic acid (**4**) 5

Figure S10. The MS^1 spectrum of lauric acid (**5**) 5

Figure S11. The MS^2 spectrum of lauric acid (**5**) 6

Figure S12. The MS^1 spectrum of myristic acid (**6**) 6

Figure S13. The MS^2 spectrum of myristic acid (**6**) 7

Figure S14. The MS^1 spectrum of tributyl citrate (**7**) 7

Figure S15. The MS^2 spectrum of tributyl citrate (**7**) 8

Figure S16. The MS^1 spectrum of ethyl myristate (**8**) 8

Figure S17. The MS^2 spectrum of ethyl myristate (**8**) 9

Figure S18. The MS^1 spectrum of stearic acid (**9**) 9

Figure S19. The MS^2 spectrum of stearic acid (**9**) 10

Figure S20. The MS^1 spectrum of pentadecanoic acid (**10**) 10

Figure S21. The MS^2 spectrum of pentadecanoic acid (**10**) 11

Figure S22. The MS^1 spectrum of oleic acid (**11**) 11

Figure S23. The MS^2 spectrum of oleic acid (**11**) 12

Figure S24. The MS^1 spectrum of trans-10-heptadecenoic acid (**12**) 12

Figure S25. The MS^2 spectrum of trans-10-heptadecenoic acid (**12**) 13

Figure S26. The MS^1 spectrum of palmitoleic acid (**13**) 13

Figure S27. The MS^2 spectrum of palmitoleic acid (**13**) 14

Figure S28. The MS^1 spectrum of undecanoic acid (**14**) 14

Figure S29. The MS^2 spectrum of undecanoic acid (**14**) 15

Figure S30. The MS^1 spectrum of linoleic Acid (**15**)....15
Figure S31. The MS^2 spectrum of linoleic Acid (**15**)....16
Figure S32. The MS^1 spectrum of (E)-6-hydroxyoctadec-4-enoic acid (**16**)....16
Figure S33. The MS^2 spectrum of (E)-6-hydroxyoctadec-4-enoic acid (**16**)....17
Figure S34. The MS^1 spectrum of 13-hydroxy-9*Z*,11*E*,15*Z*-octadecatrienoic acid (**17**)....17
Figure S35. The MS^2 spectrum of 13-hydroxy-9*Z*,11*E*,15*Z*-octadecatrienoic acid (**17**)....18
Figure S36. The MS^1 spectrum of 16-hydroxyhexadecanoic acid (**18**)....18
Figure S37. The MS^2 spectrum of 16-hydroxyhexadecanoic acid (**18**)....19
Figure S38. The MS^1 spectrum of tributyl phosphate (**19**)....19
Figure S39. The MS^2 spectrum of tributyl phosphate (**19**)....20
Figure S40. The MS^1 spectrum of dibutyl phosphate (**20**)....20
Figure S41. The MS^2 spectrum of dibutyl phosphate (**20**)....21
Figure S42. The MS^1 spectrum of triphenylphosphine oxide (**21**)....21
Figure S43. The MS^2 spectrum of triphenylphosphine oxide (**21**)....22
Figure S44. The MS^1 spectrum of triethyl phosphate (**22**)....22
Figure S45. The MS^2 spectrum of triethyl phosphate (**22**)....23
Figure S46. The MS^1 spectrum of hexadecanamide (**23**)....23
Figure S47. The MS^2 spectrum of hexadecanamide (**23**)....24
Figure S48. The MS^1 spectrum of 4,6-dimethyl-2-hydroxypyrimidine (**24**)....24
Figure S49. The MS^2 spectrum of 4,6-dimethyl-2-hydroxypyrimidine (**24**)....25
Figure S50. The MS^1 spectrum of cinchophen (**25**)....25
Figure S51. The MS^2 spectrum of cinchophen (**25**)....26
Figure S52. The MS^1 spectrum of N, N-dicyclohexylurea (**26**)....26
Figure S53. The MS^2 spectrum of N, N-dicyclohexylurea (**26**)....27
Figure S54. The MS^1 spectrum of dodecyl sulfate (**27**)....27
Figure S55. The MS^2 spectrum of dodecyl sulfate (**27**)....28
Figure S56. The MS^1 spectrum of myristyl sulfate (**28**)....28
Figure S57. The MS^2 spectrum of myristyl sulfate (**28**)....29
Figure S58. The MS^1 spectrum of 4-dodecylbenzenesulfonic acid (**29**)....29
Figure S59. The MS^2 spectrum of 4-dodecylbenzenesulfonic acid (**29**)....30
Figure S60. The MS^1 spectrum of 2,4-di-tert-butylphenol (**30**)....30

Figure S61. The MS^2 spectrum of 2,4-di-tert-butylphenol (**30**)..31
Figure S62. The MS^1 spectrum of dibutyl phthalate (**31**) 31
Figure S63. The MS^2 spectrum of dibutyl phthalate (**31**) 32
Figure S64. The MS^1 spectrum of 2,2'-methylenebis(4-methyl-6-tert-butylphenol) (**32**)..............32
Figure S65. The MS^2 spectrum of 2,2'-methylenebis(4-methyl-6-tert-butylphenol) (**32**)..............33
Figure S66. The MS^1 spectrum of abietic acid (**33**)..33
Figure S67. The MS^2 spectrum of abietic acid (**33**)..34
Figure S68. The MS^1 spectrum of tretinoin (**34**)..34
Figure S69. The MS^2 spectrum of tretinoin (**34**)..35
Figure S70. The MS^1 spectrum of flavone (**35**) 35
Figure S71. The MS^2 spectrum of flavone (**35**) 36
Figure S72. The MS^1 spectrum of bis(2-butoxyethyl) ethe (**36**)..36
Figure S73. The MS^2 spectrum of bis(2-butoxyethyl) ethe (**36**)..37
Figure S74. The MS^1 spectrum of trenbolone (**37**) 37
Figure S75. The MS^2 spectrum of trenbolone (**37**) 38
Figure S76. The chemical structures of compounds (**1-37**)...39-40

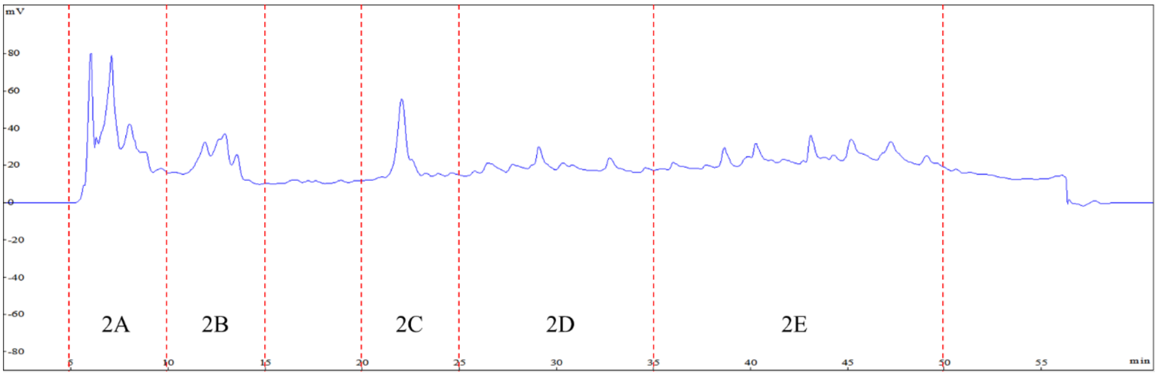

Figure S1. The subfraction of the n-butanol extraction P2 from bleeding sap

(Mobile phase: MeOH/Acid-H_2O, 10%-100%; column: CT-30 Fuji-C_{18}, 280 × 30 mm, 10 µm;

flow rate: 10 mL/min; detector wavelength: 254 nm)

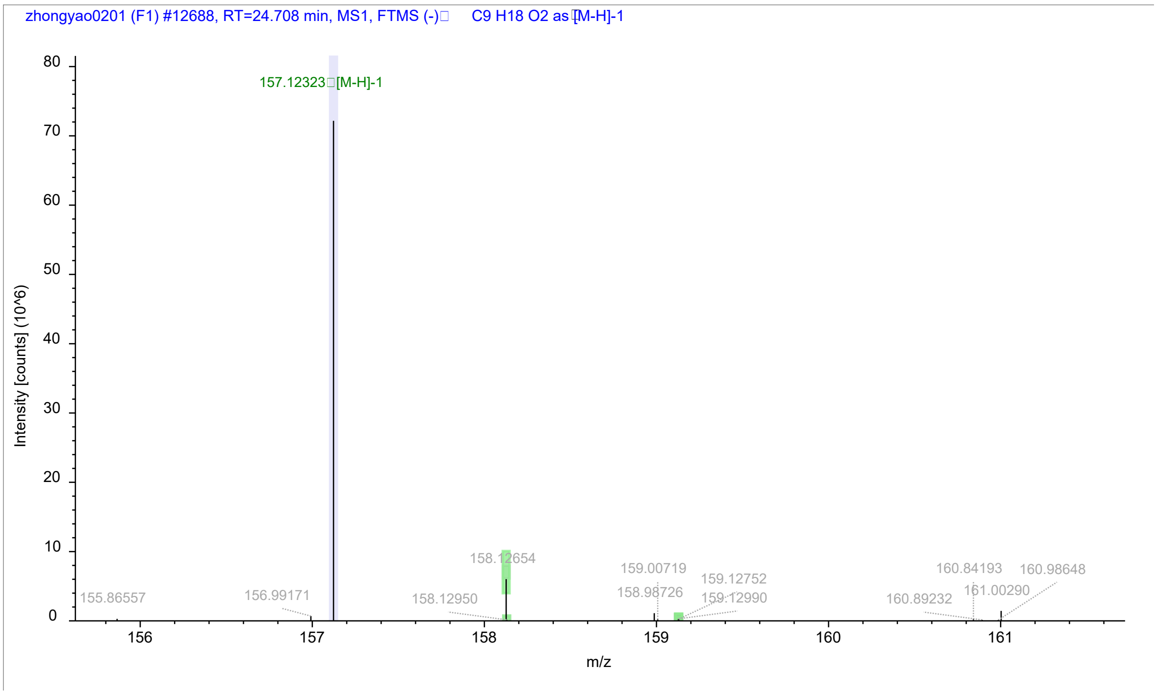

Figure S2. The MS^1 spectrum of nonanoic acid (**1**)

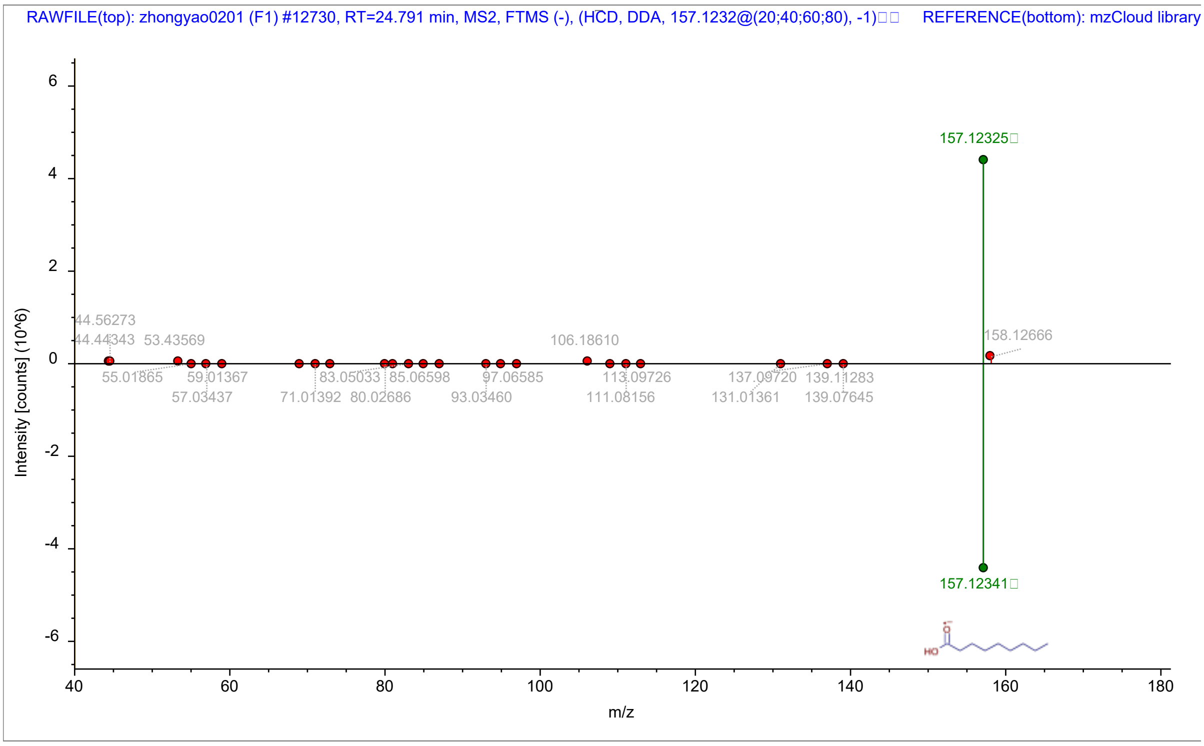

Figure S3. The MS^2 spectrum of nonanoic acid (**1**)

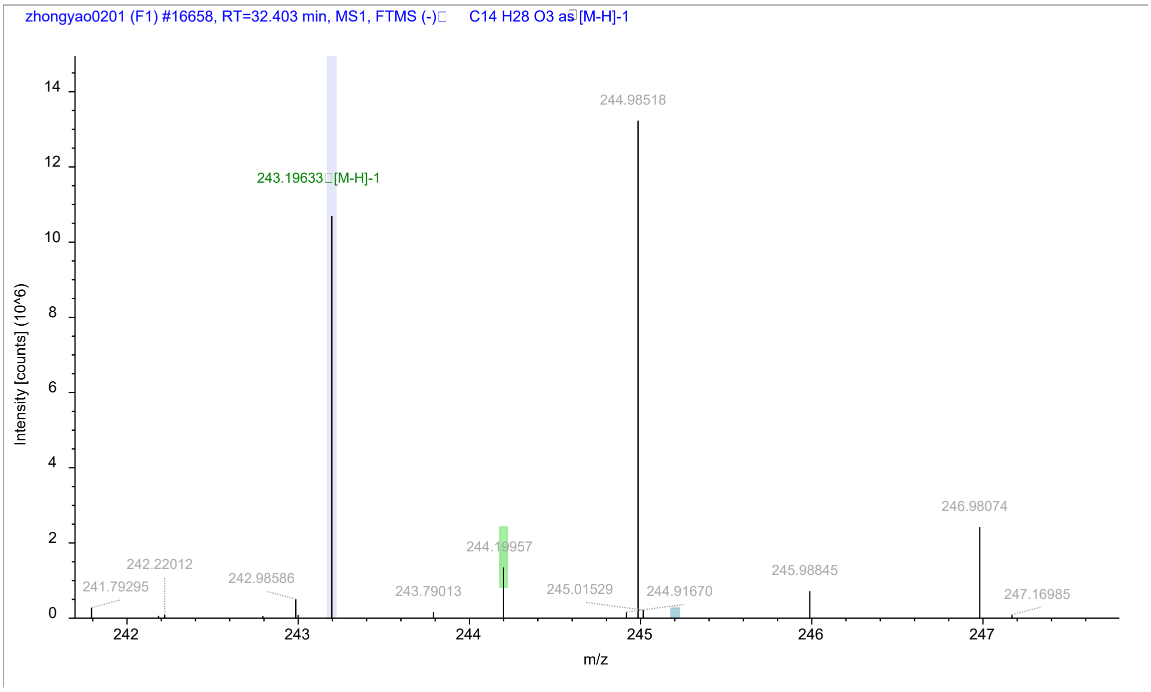

Figure S4. The MS^1 spectrum of 3-hydroxy myristic acid (**2**)

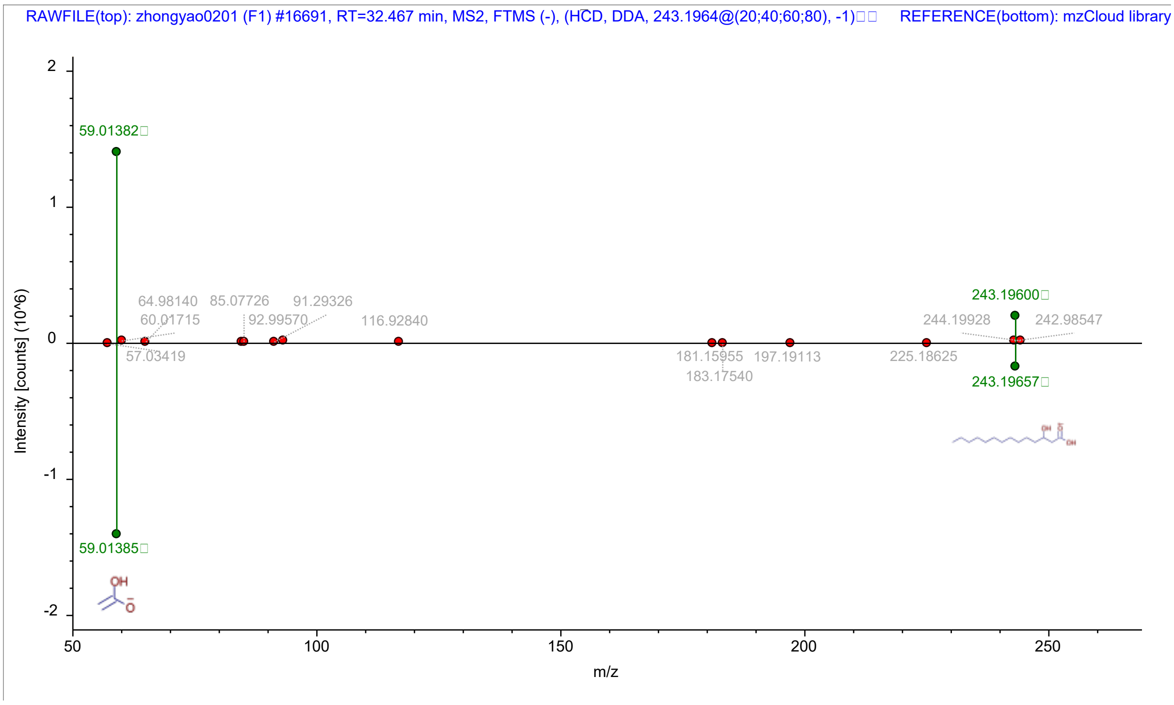

Figure S5. The MS^2 spectrum of 3-hydroxy myristic acid (**2**)

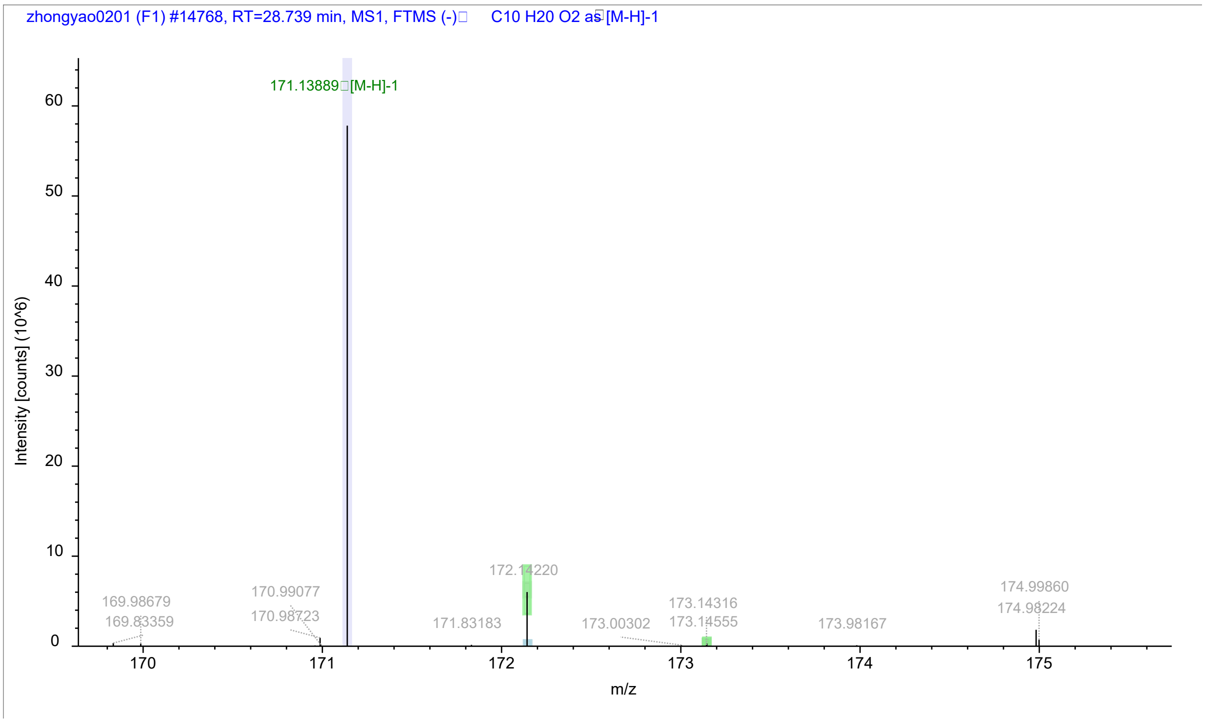

Figure S6. The MS^1 spectrum of decanoic acid (**3**)

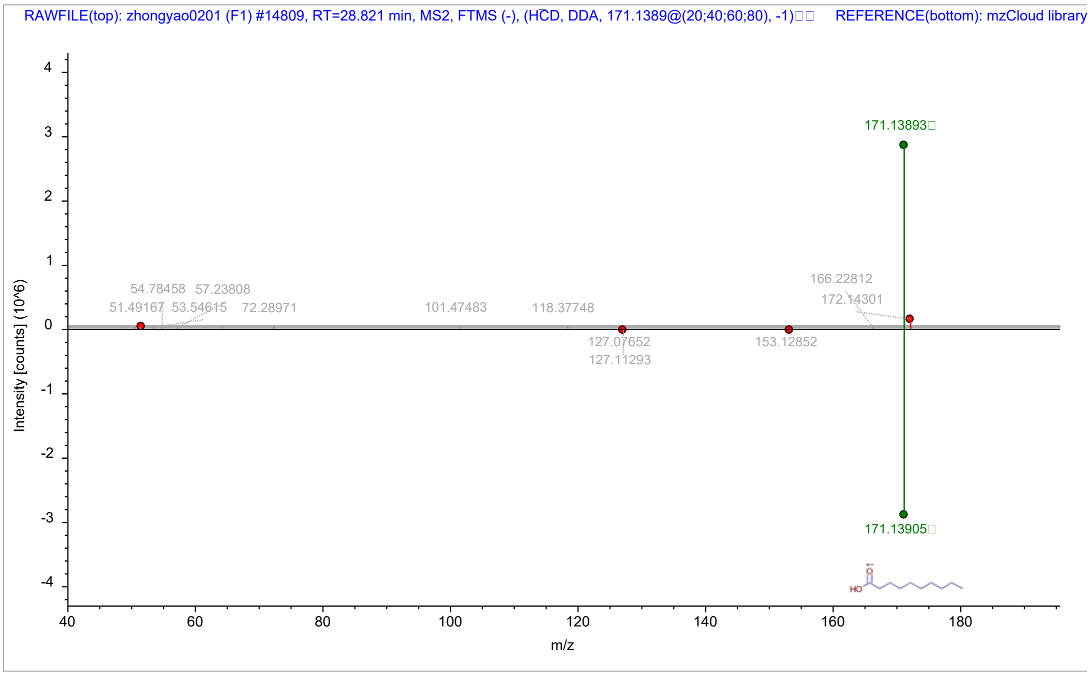

Figure S7. The MS^2 spectrum of decanoic acid (**3**)

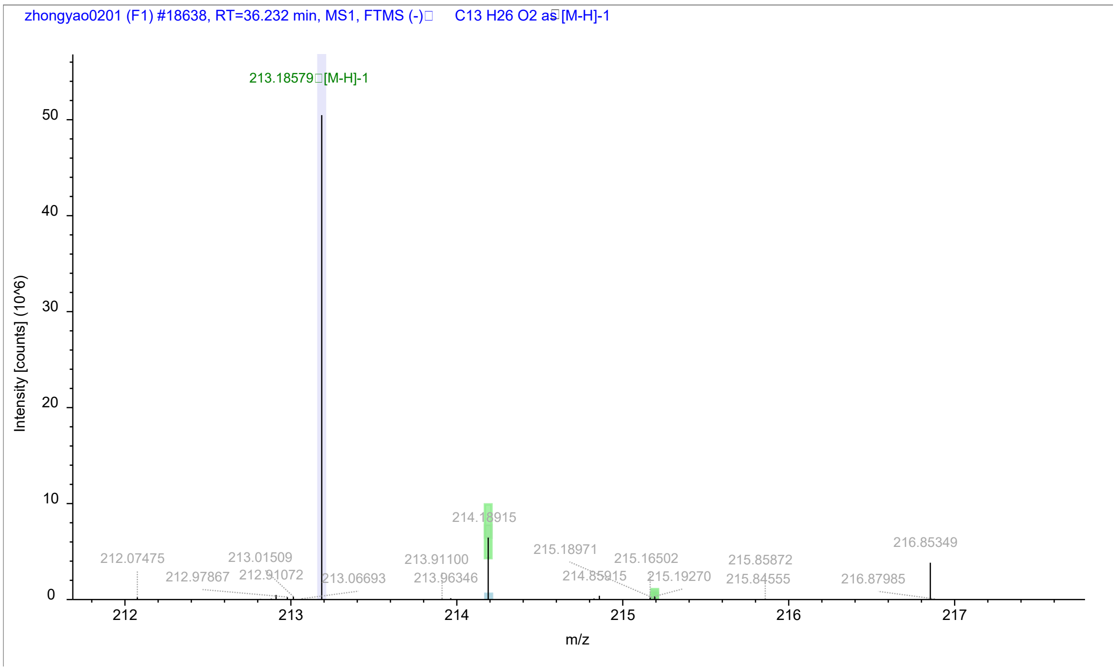

Figure S8. The MS^1 spectrum of tridecylic acid (**4**)

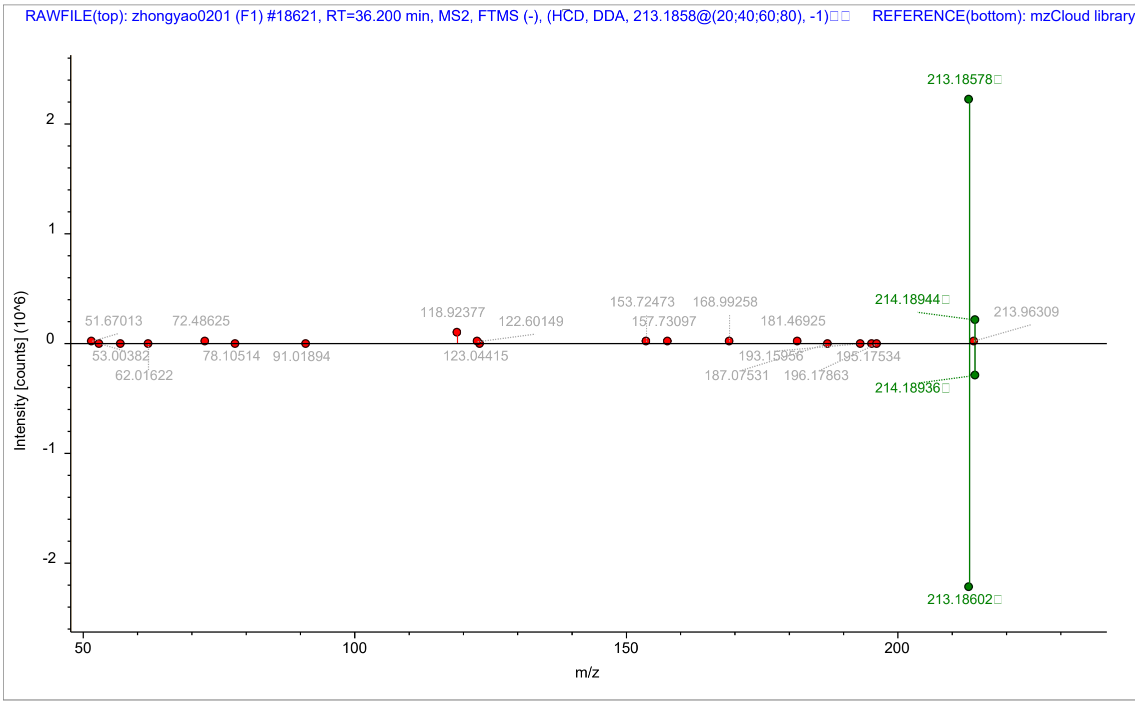

Figure S9. The MS^2 spectrum of tridecylic acid (**4**)

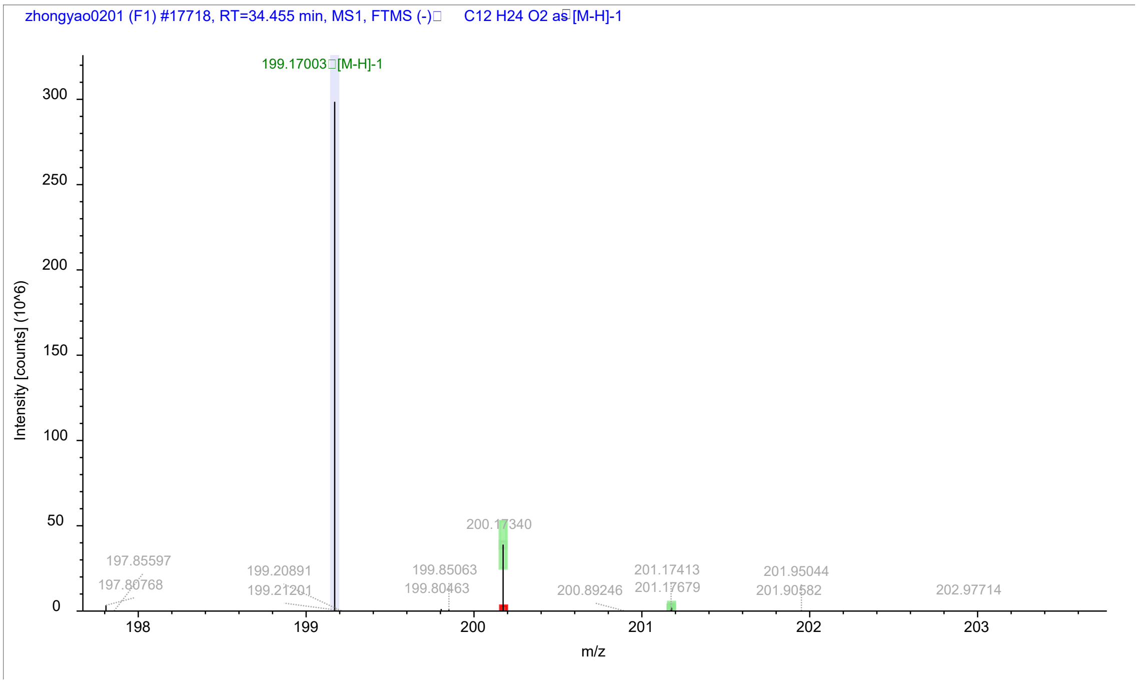

Figure S10. The MS^1 spectrum of lauric acid (**5**)

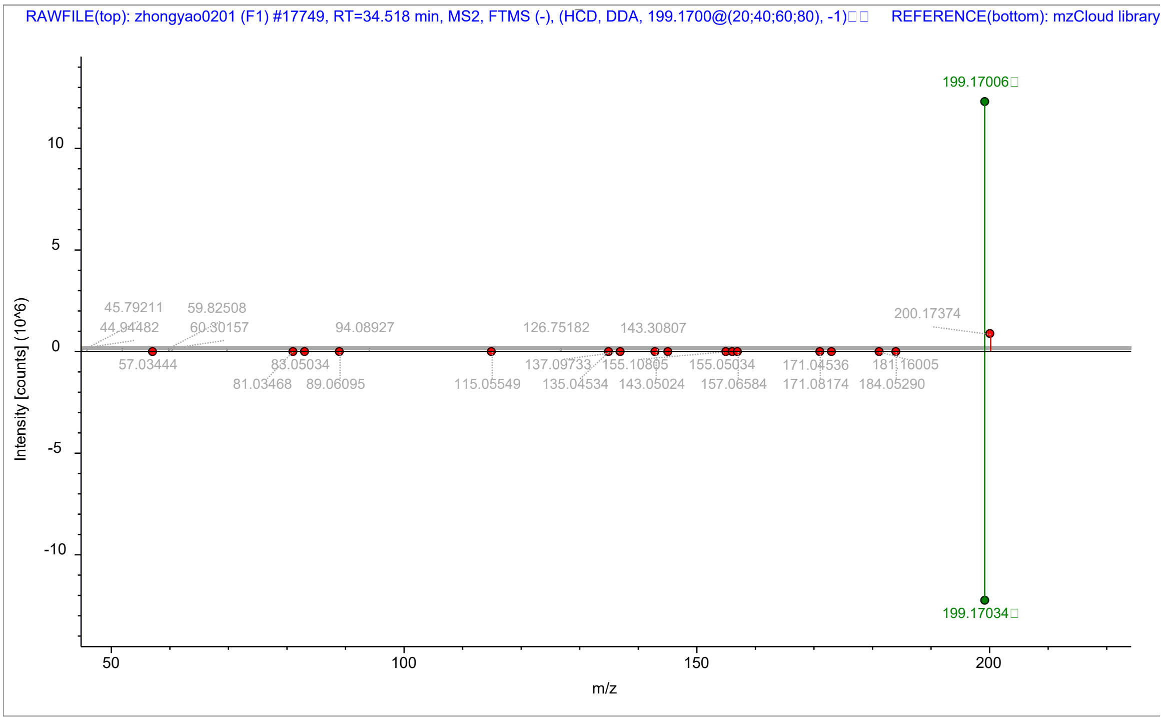

Figure S11. The MS^2 spectrum of lauric acid (**5**)

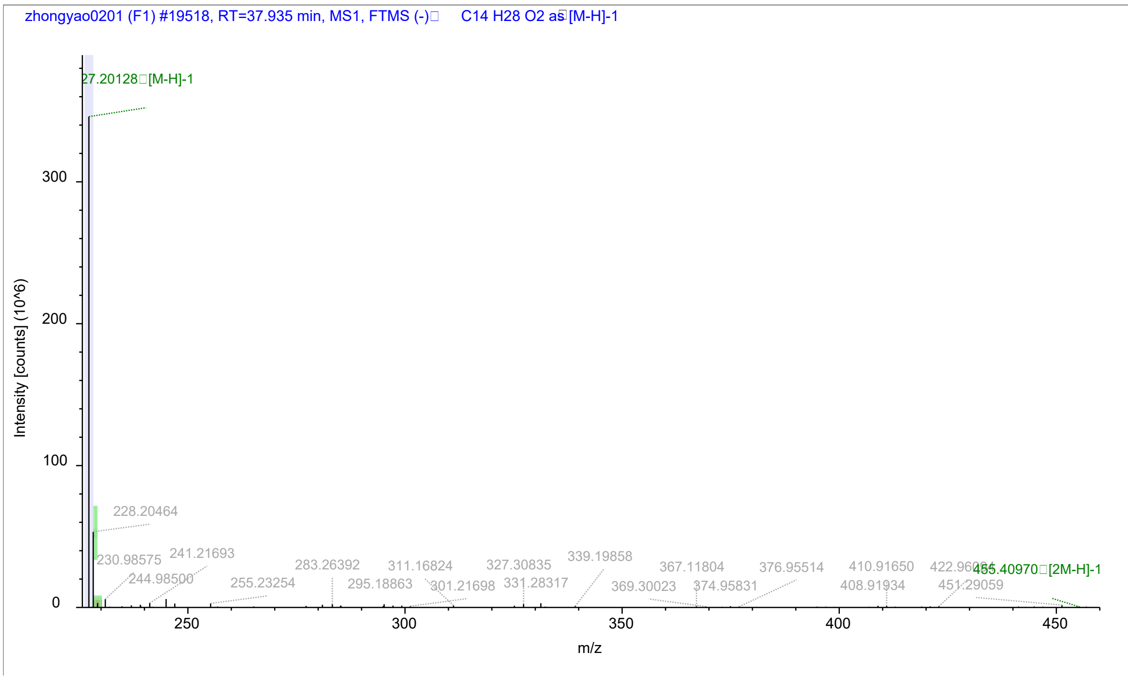

Figure S12. The MS^1 spectrum of myristic acid (**6**)

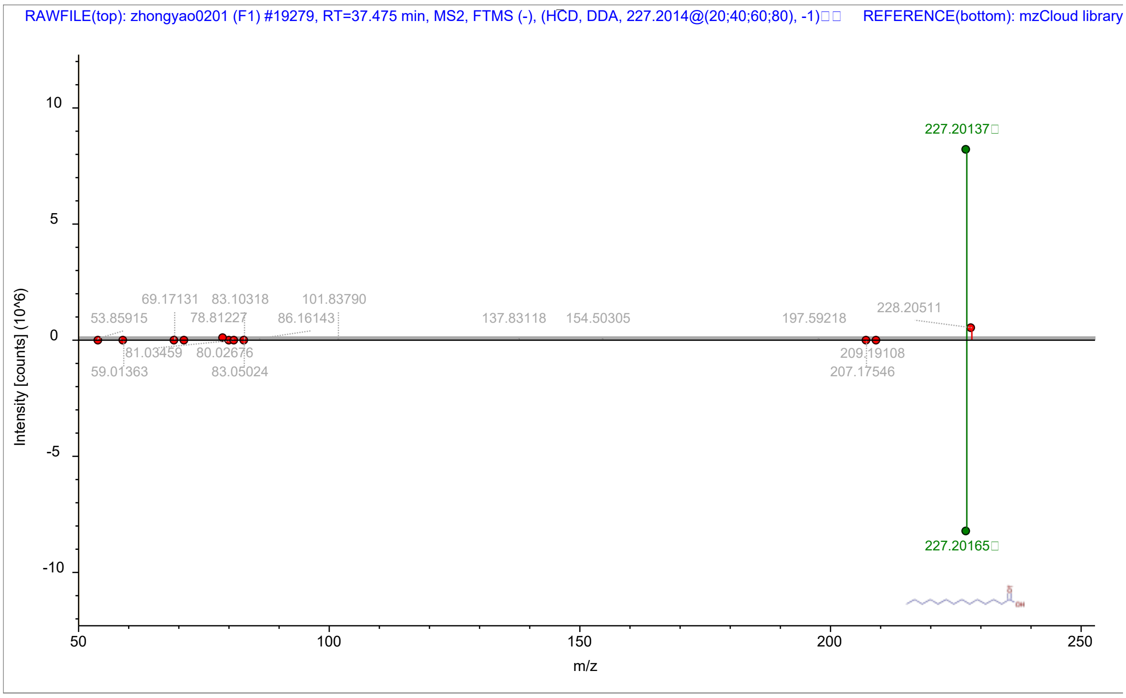

Figure S13. The MS^2 spectrum of myristic acid (**6**)

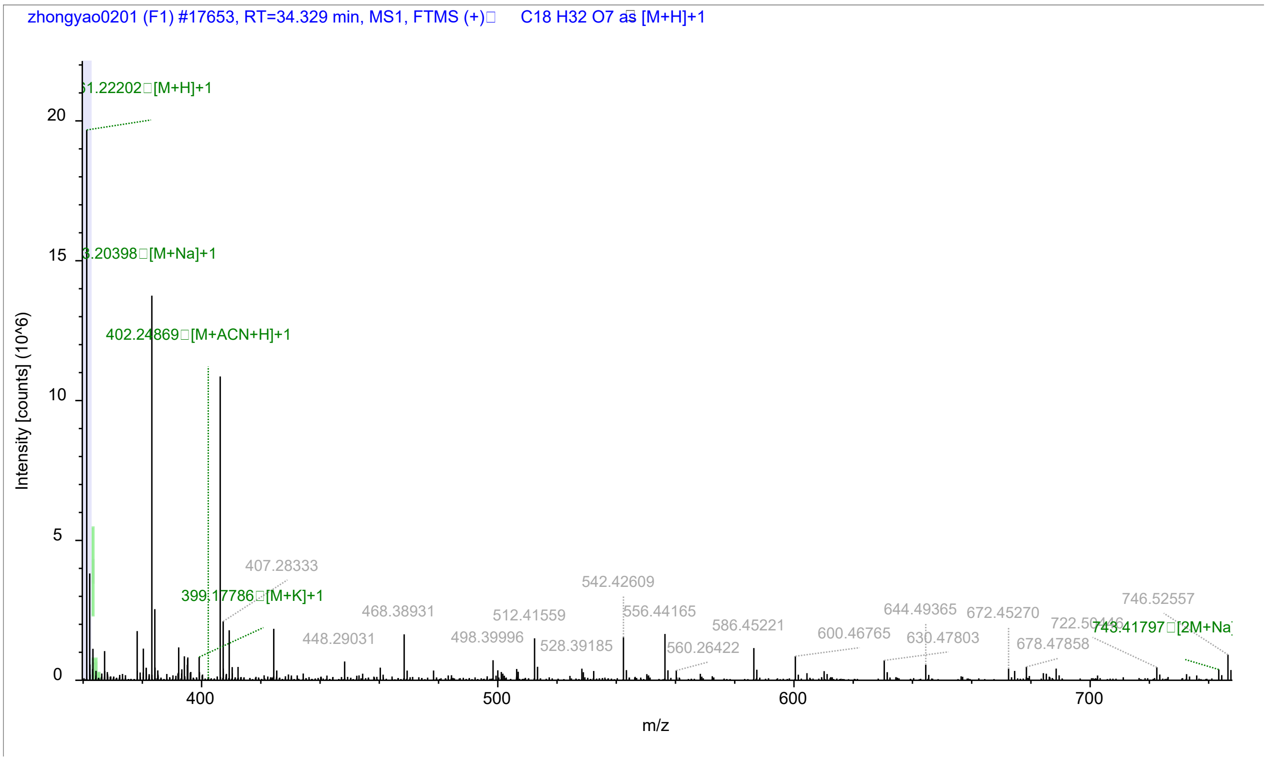

Figure S14. The MS^1 spectrum of tributyl citrate (**7**)

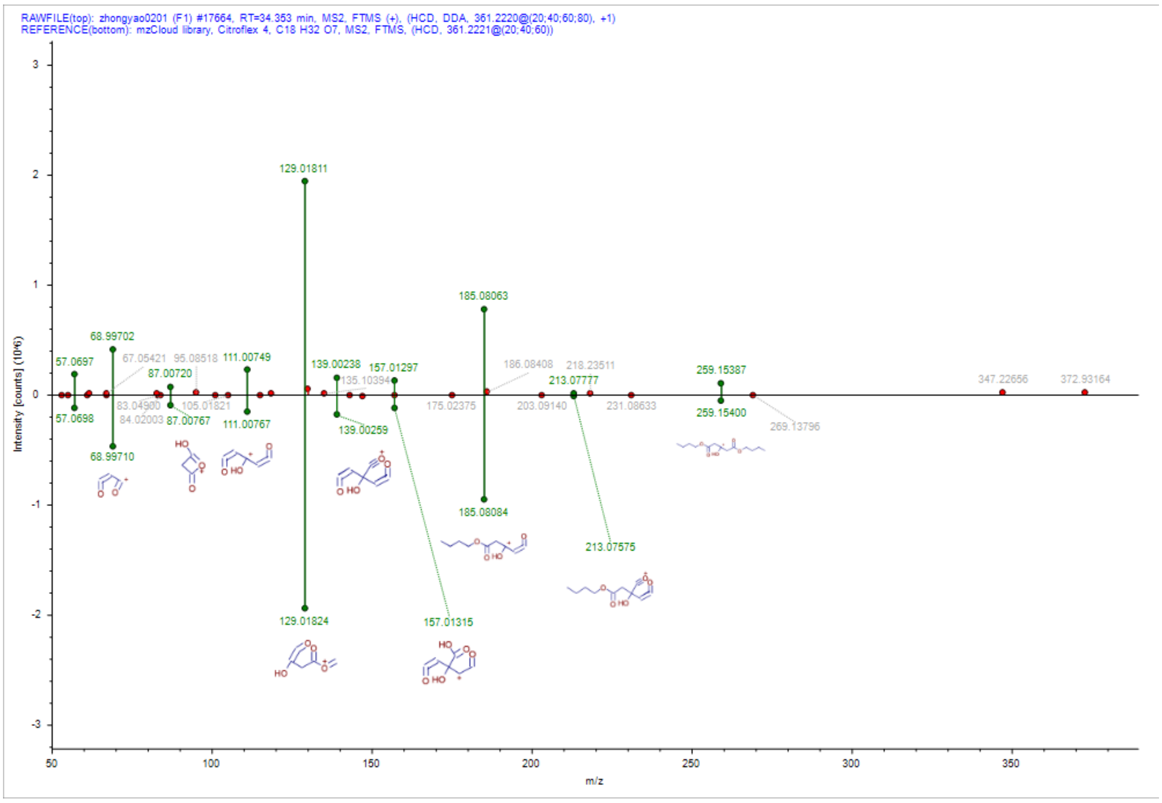

Figure S15. The MS^2 spectrum of tributyl citrate (**7**)

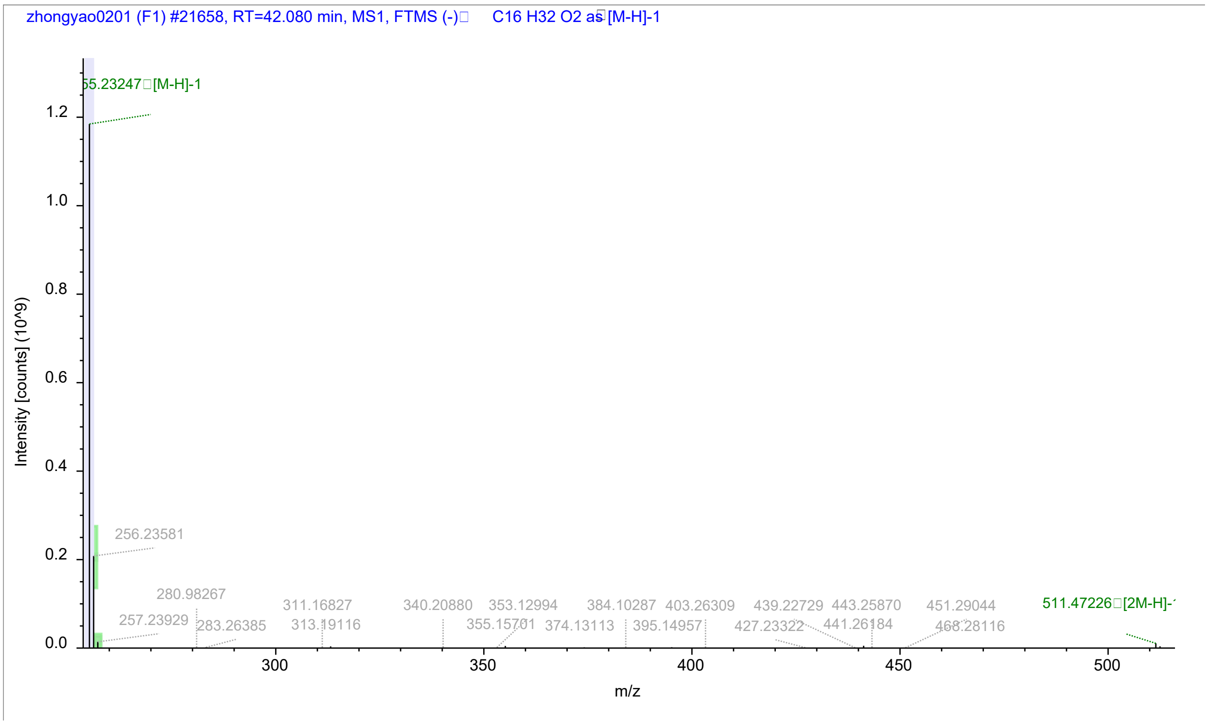

Figure S16. The MS^1 spectrum of ethyl myristate (**8**)

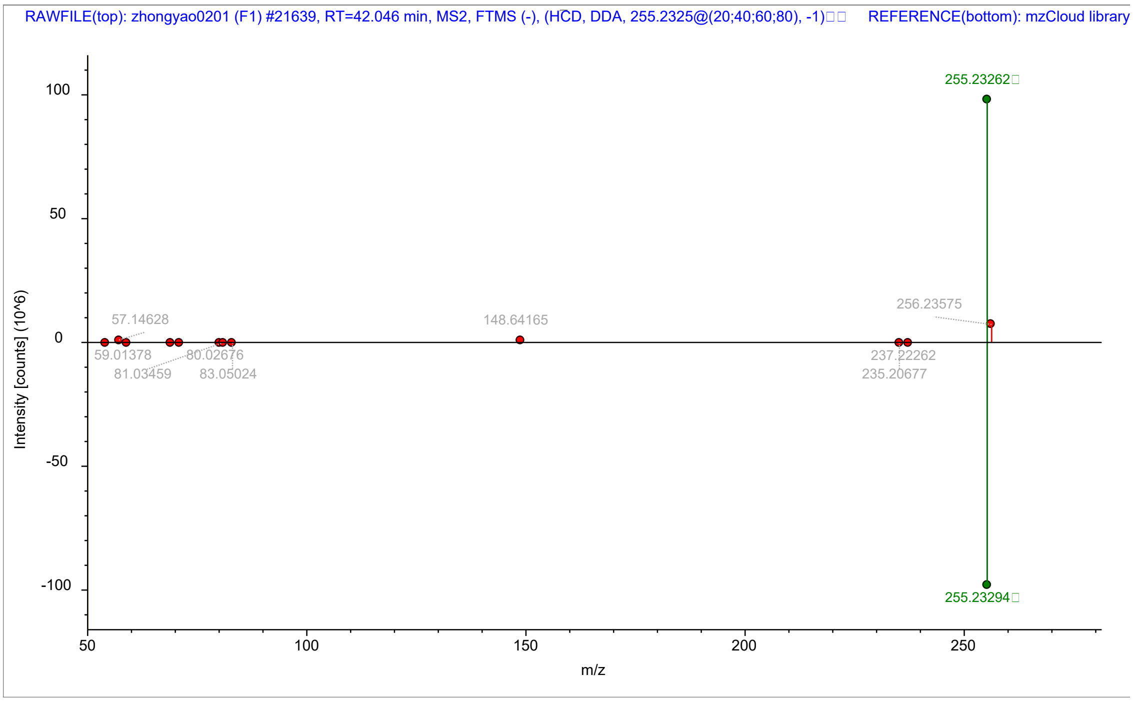

Figure S17. The MS[2] spectrum of ethyl myristate (**8**)

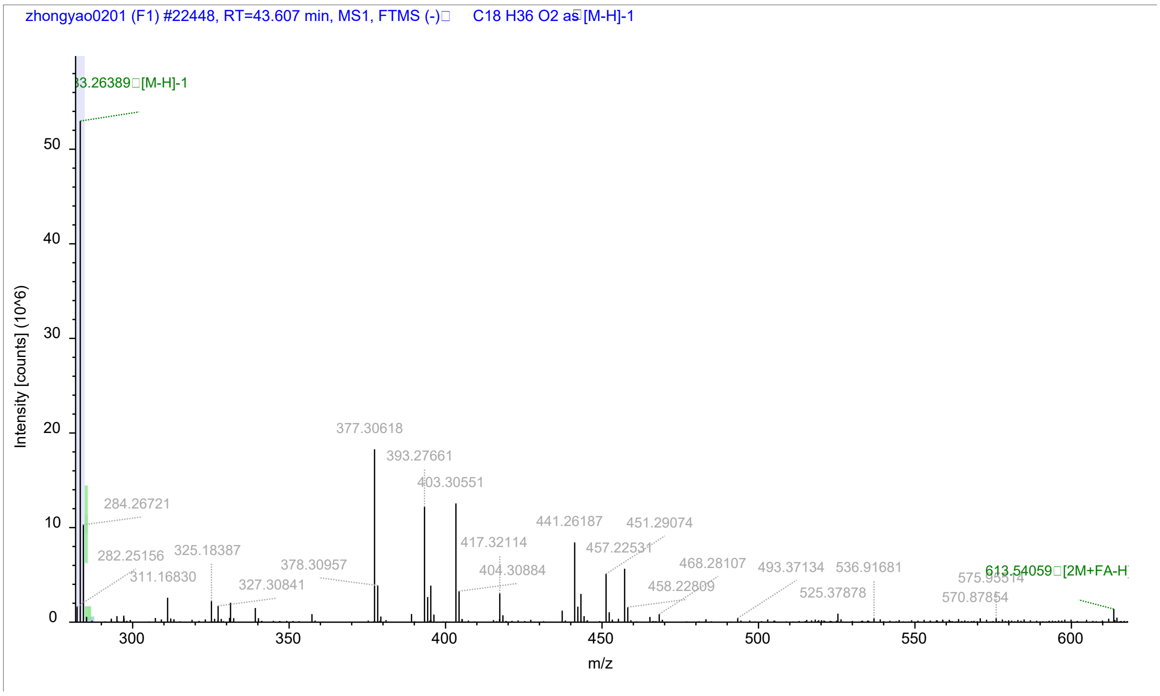

Figure S18. The MS[1] spectrum of stearic acid (**9**)

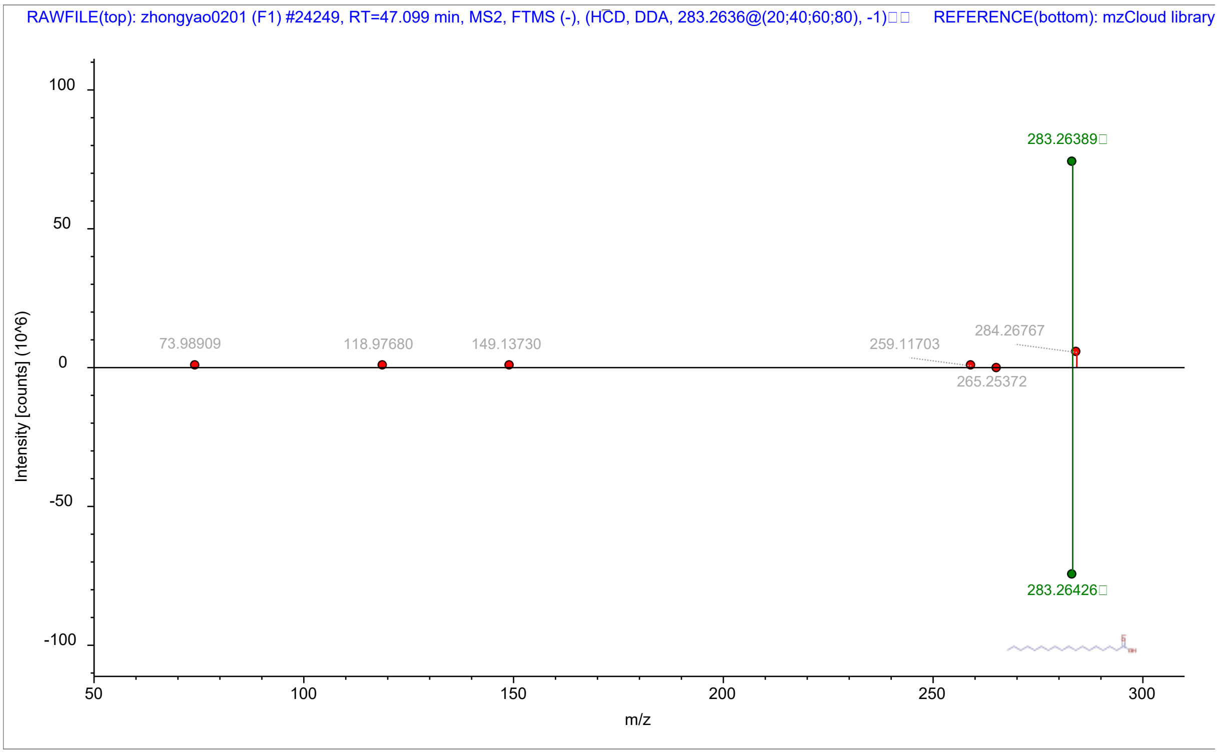

Figure S19. The MS^2 spectrum of stearic acid (**9**)

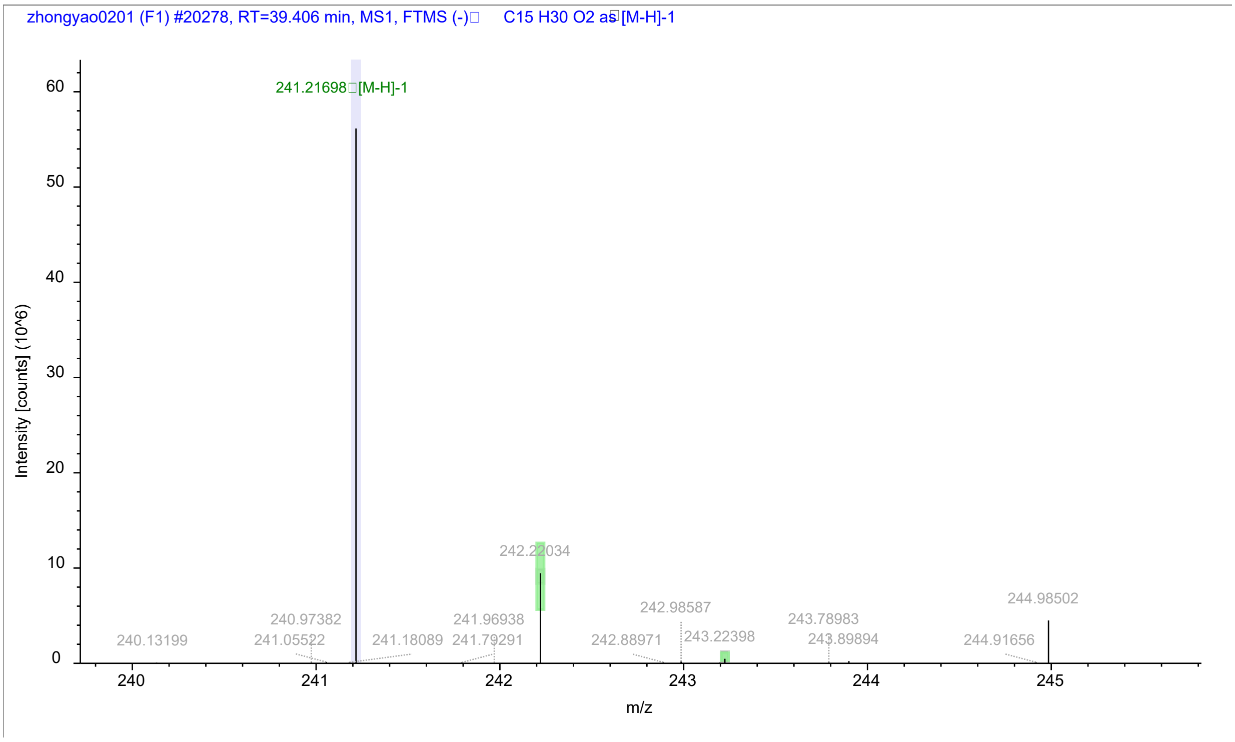

Figure S20. The MS^1 spectrum of pentadecanoic acid (**10**)

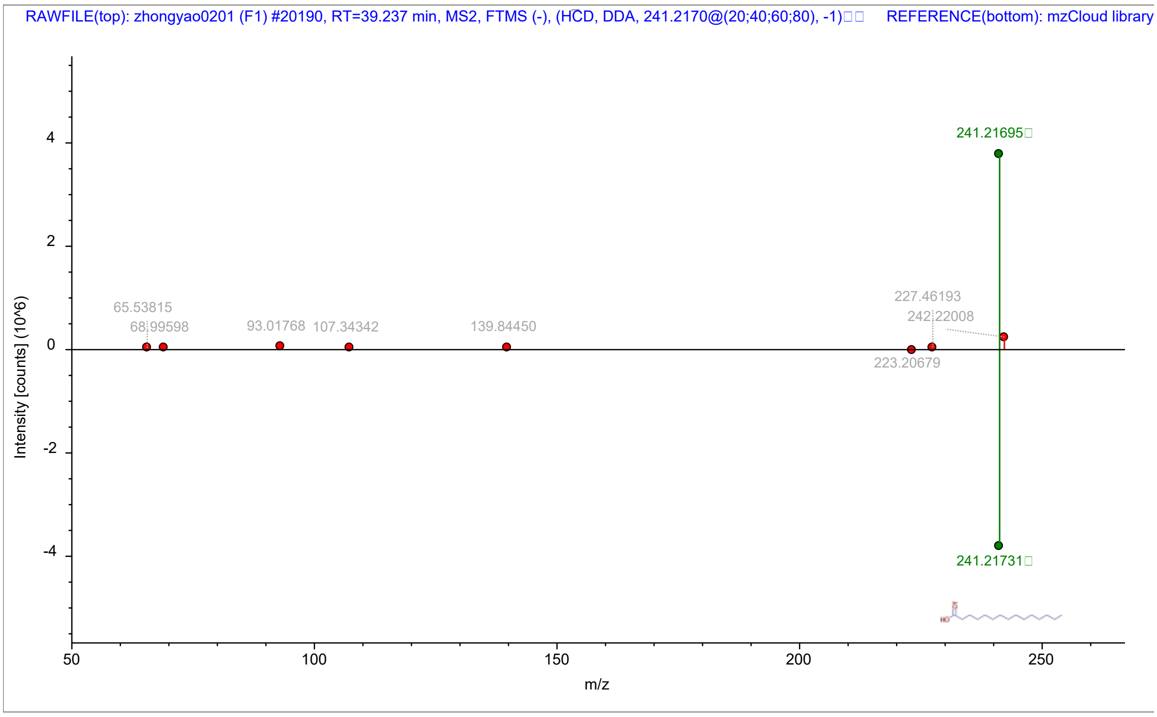

Figure S21. The MS^2 spectrum of pentadecanoic acid (**10**)

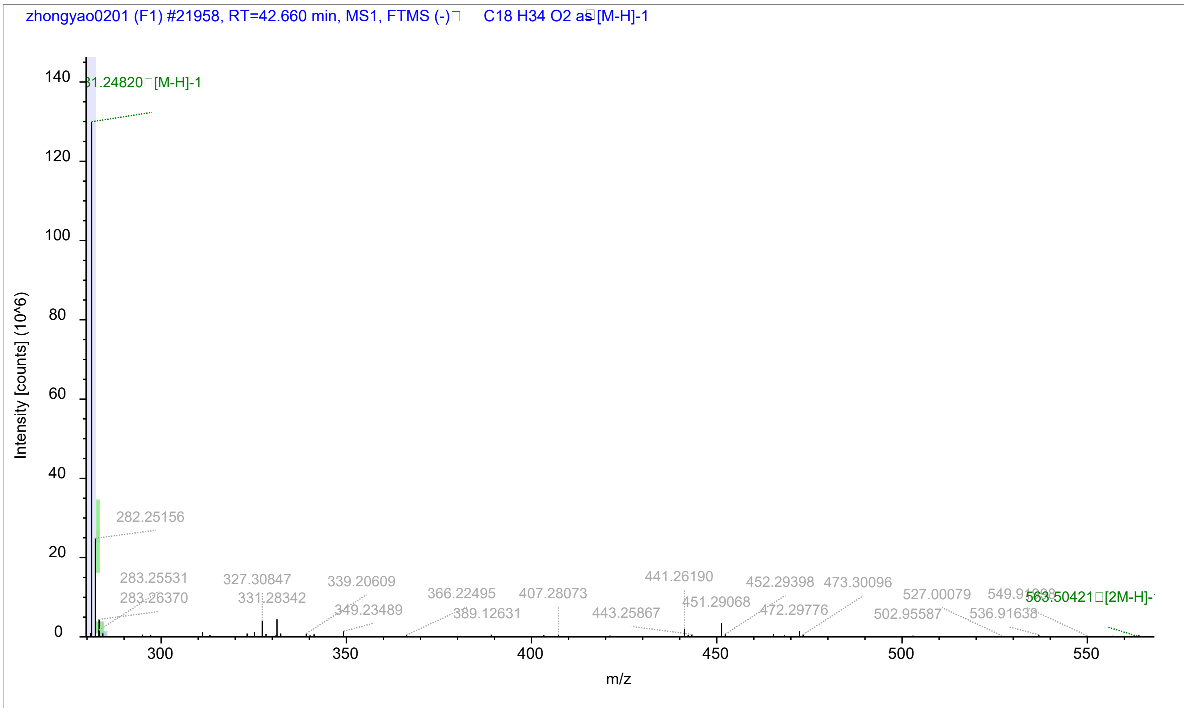

Figure S22. The MS^1 spectrum of oleic acid (**11**)

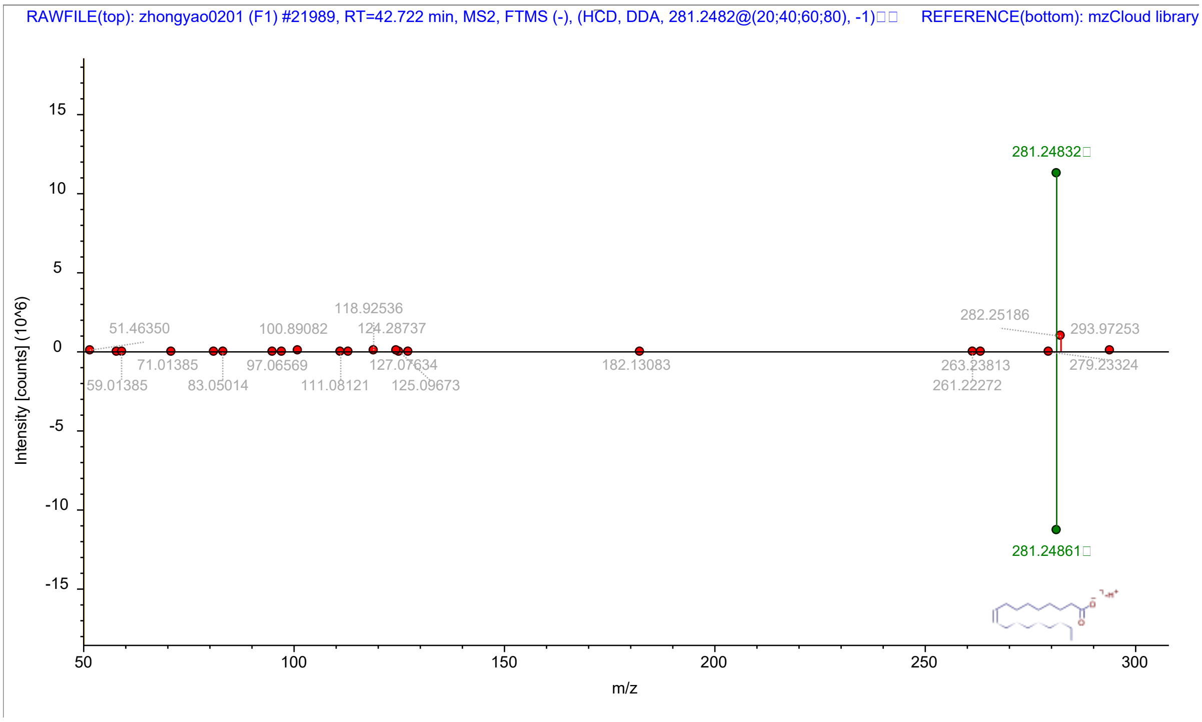

Figure S23. The MS[2] spectrum of oleic acid (**11**)

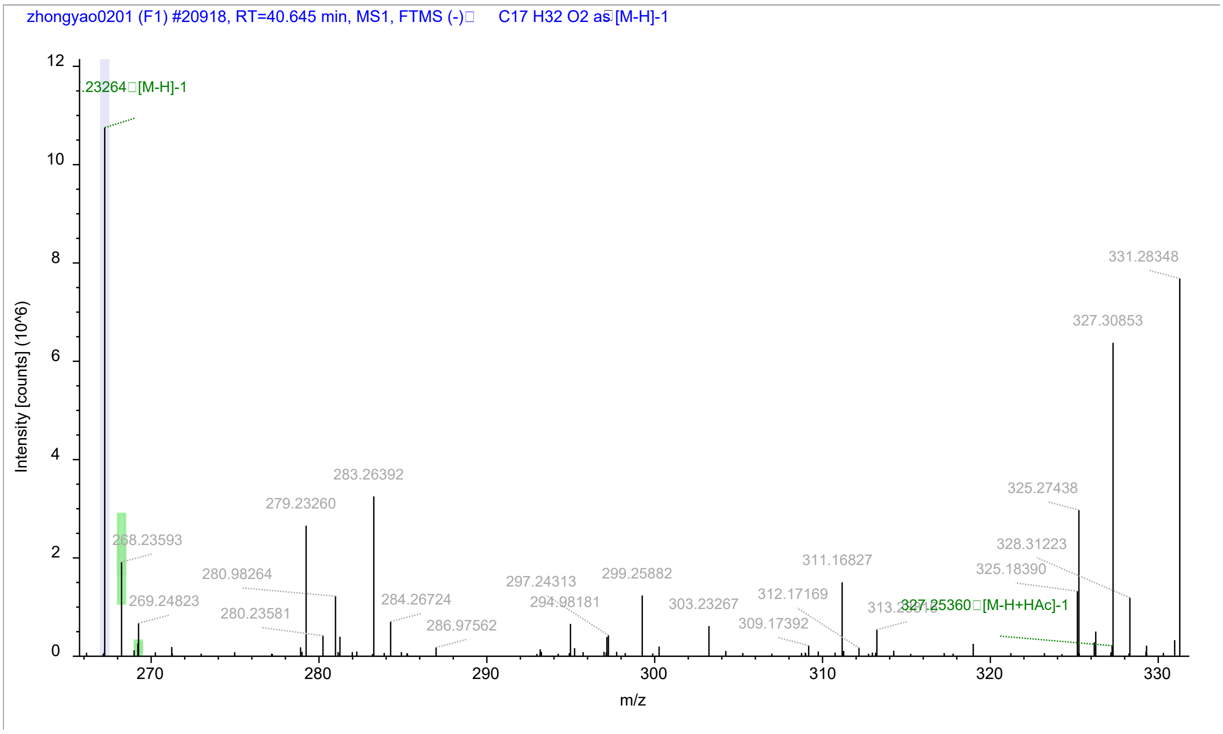

Figure S24. The MS[1] spectrum of trans-10-heptadecenoic acid (**12**)

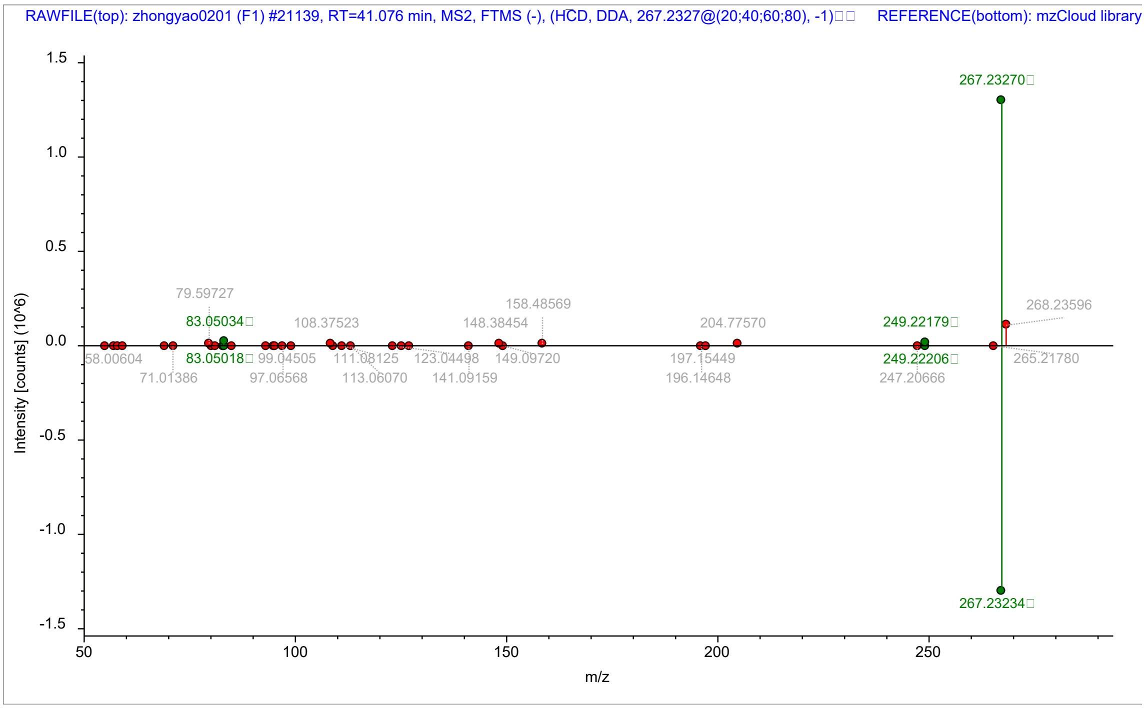

Figure S25. The MS^2 spectrum of trans-10-heptadecenoic acid (**12**)

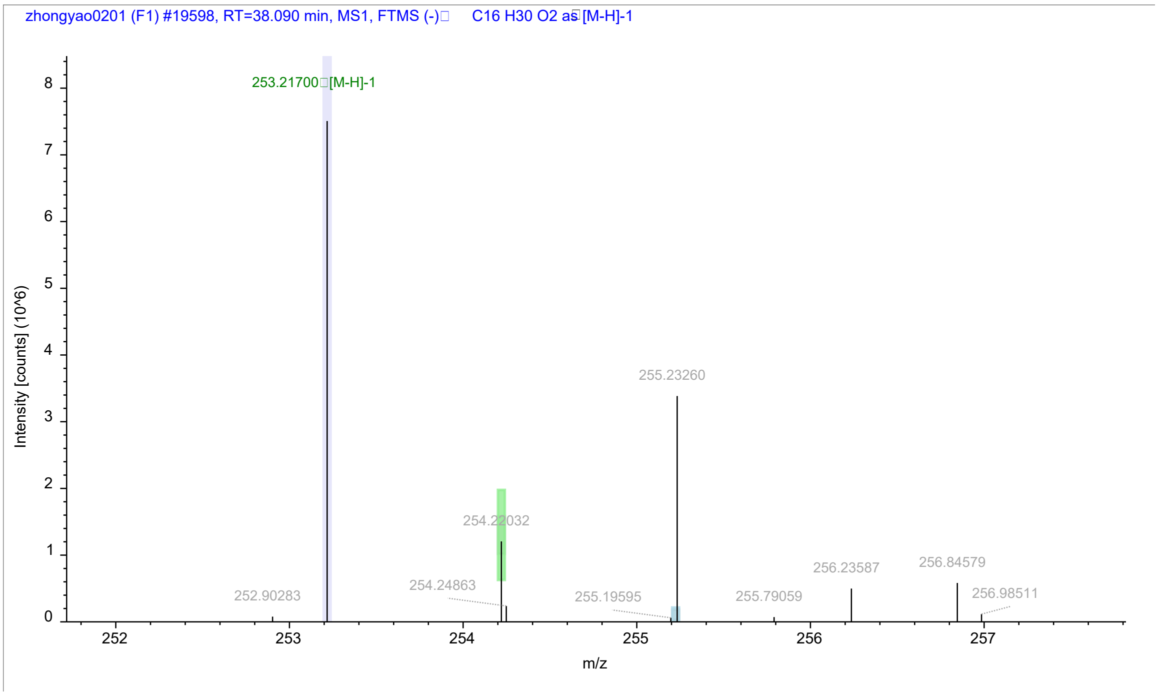

Figure S26. The MS^1 spectrum of palmitoleic acid (**13**)

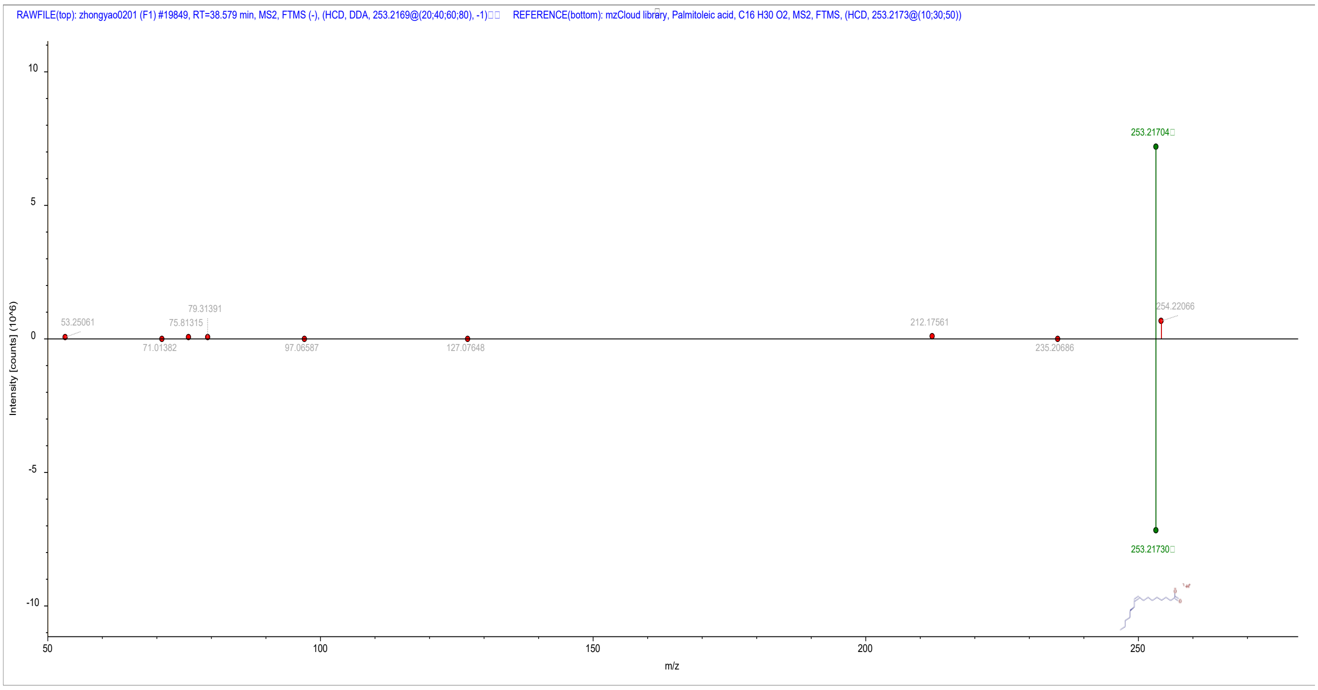

Figure S27. The MS^2 spectrum of palmitoleic acid (**13**)

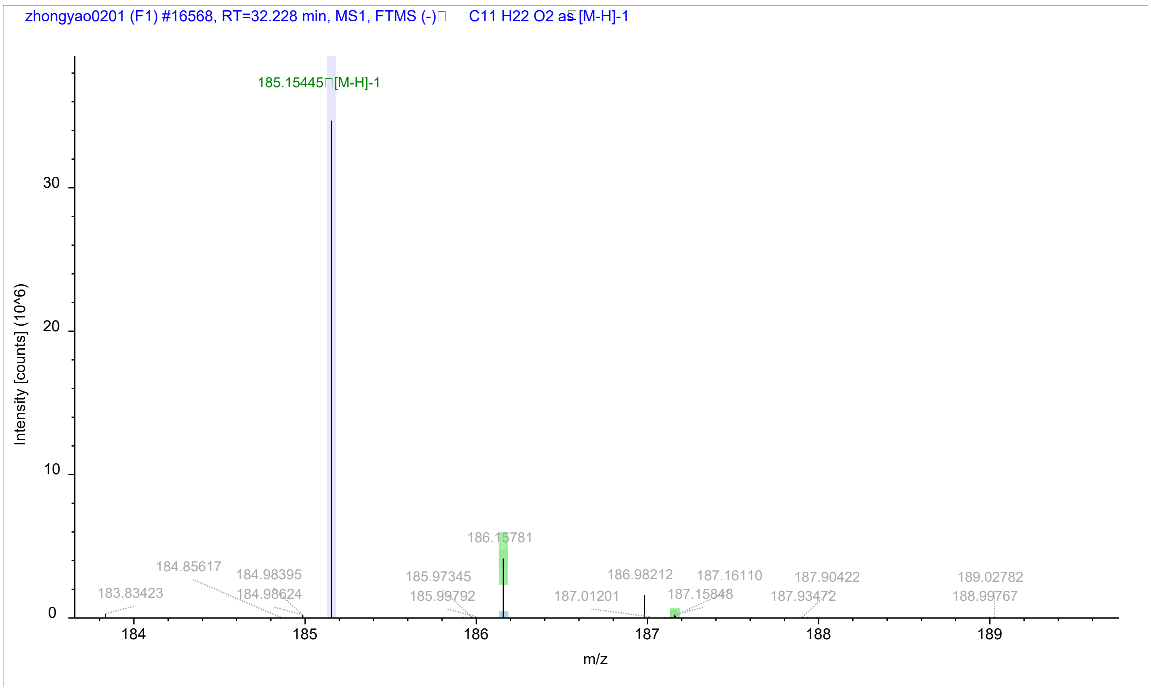

Figure S28. The MS^1 spectrum of undecanoic acid (**14**)

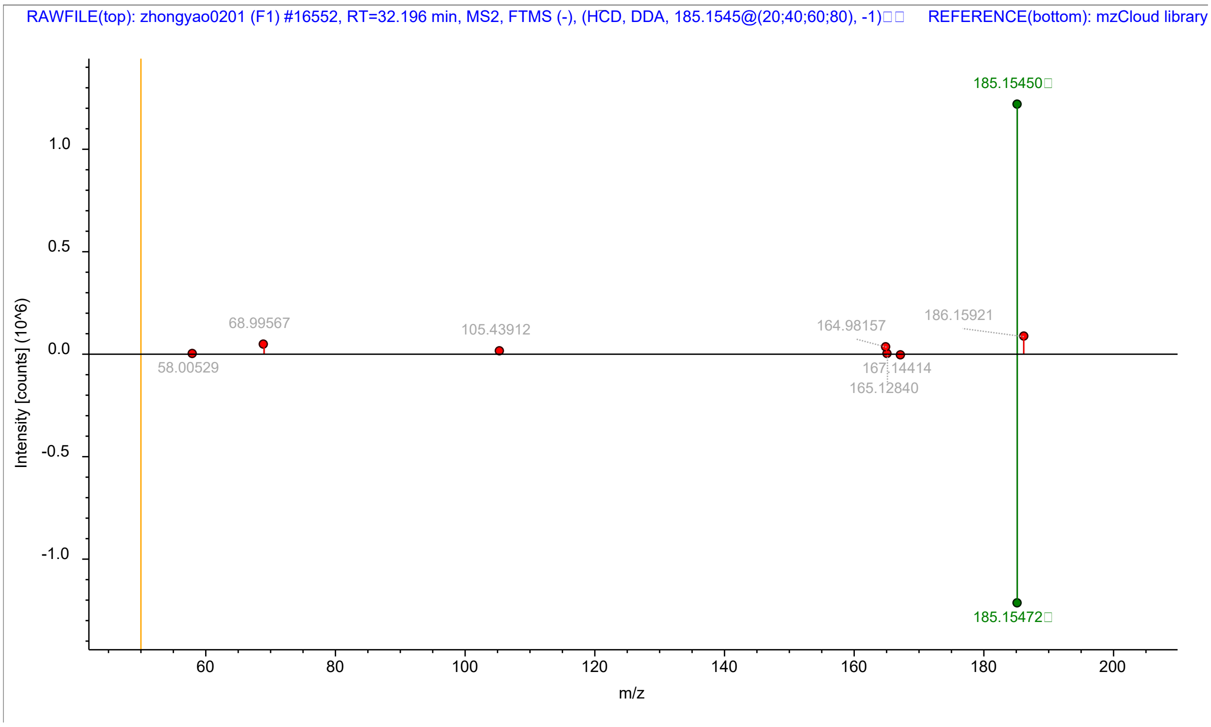

Figure S29. The MS^2 spectrum of undecanoic acid (**14**)

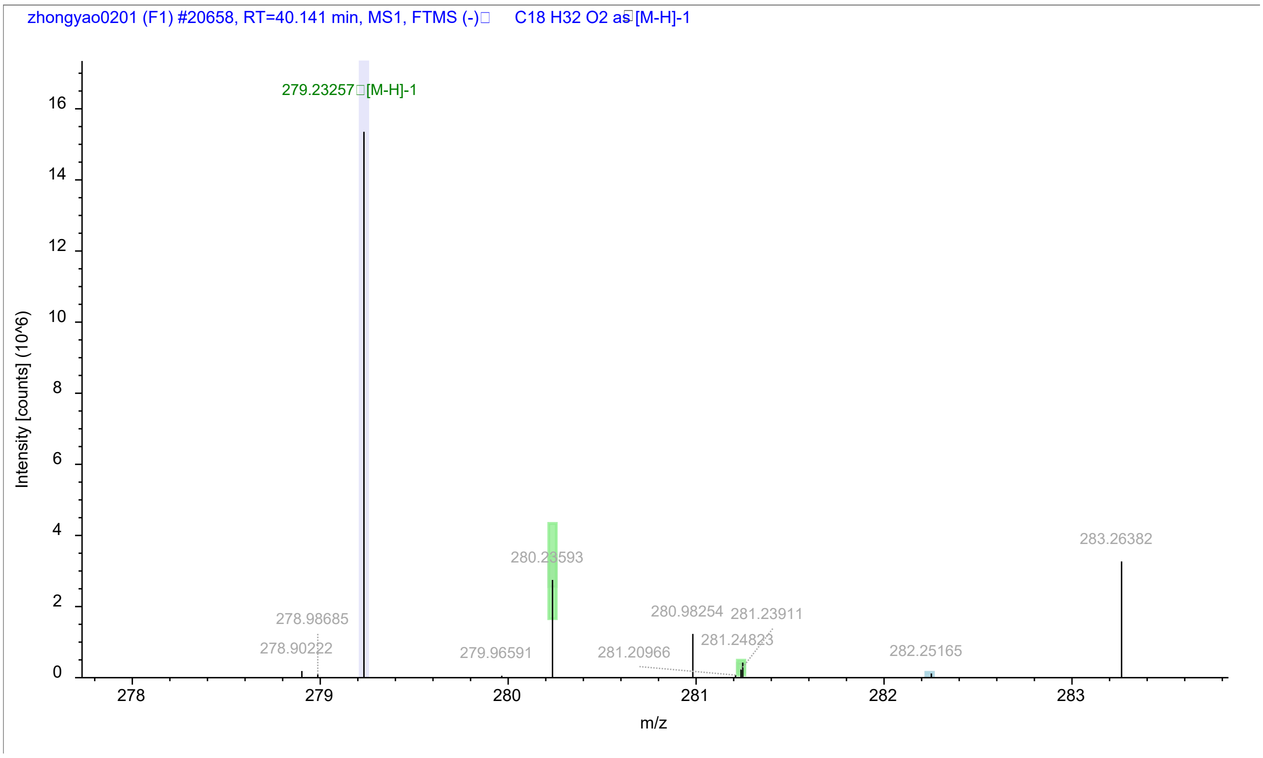

Figure S30. The MS^1 spectrum of linoleic Acid (**15**)

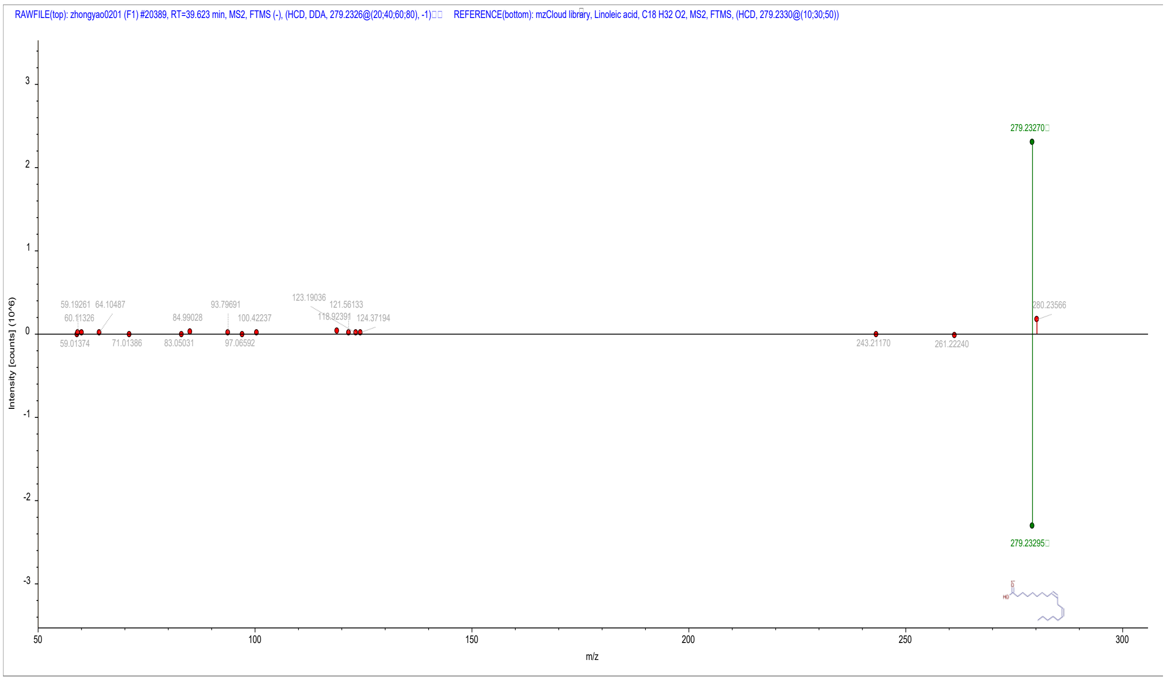

Figure S31. The MS^2 spectrum of linoleic Acid (**15**)

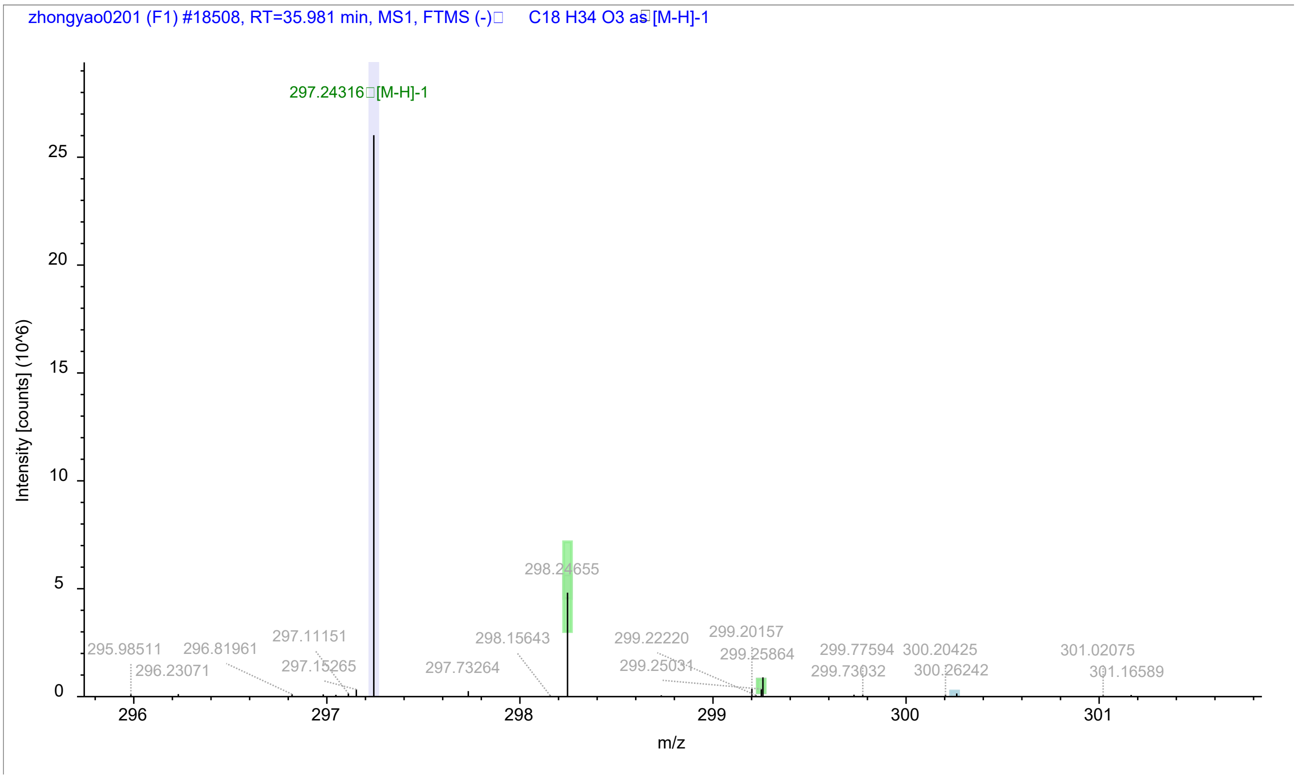

Figure S32. The MS^1 spectrum of (E)-6-hydroxyoctadec-4-enoic acid (**16**)

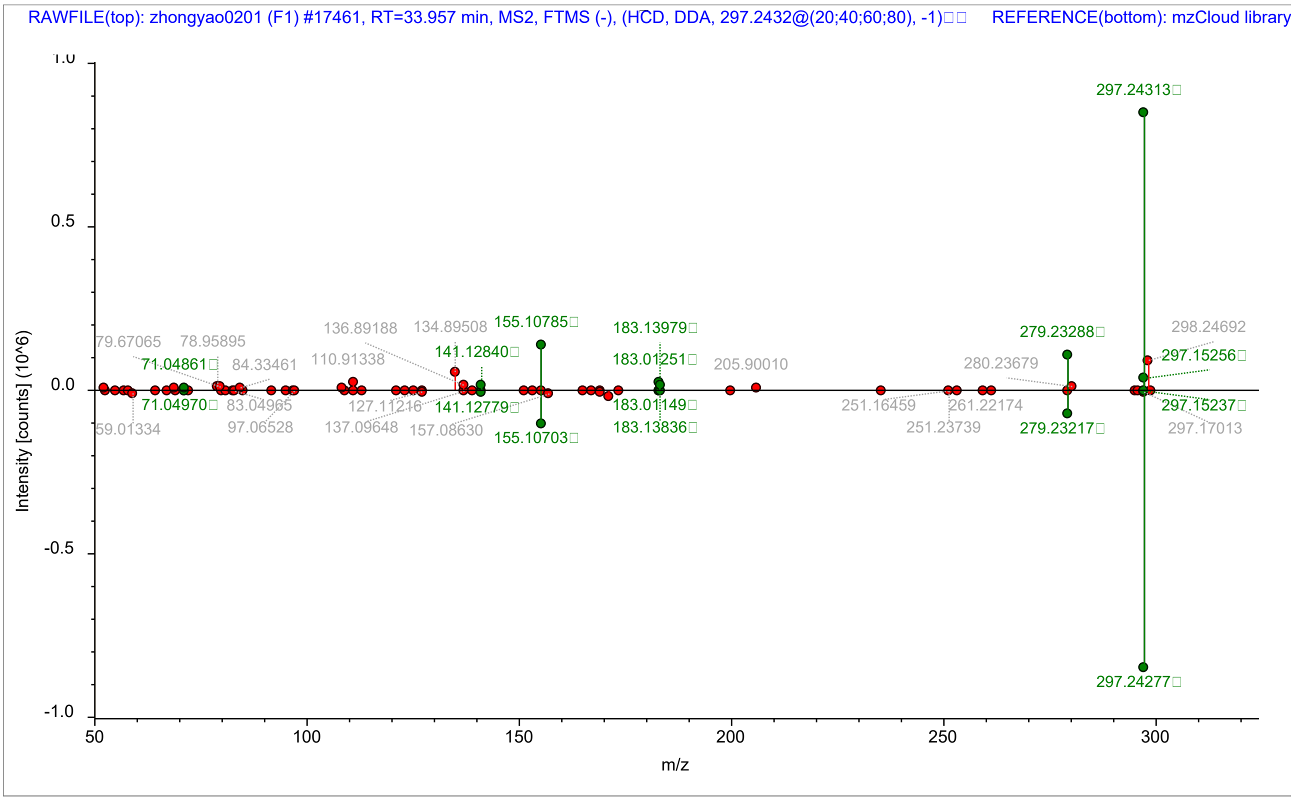

Figure S33. The MS^2 spectrum of (E)-6-hydroxyoctadec-4-enoic acid (**16**)

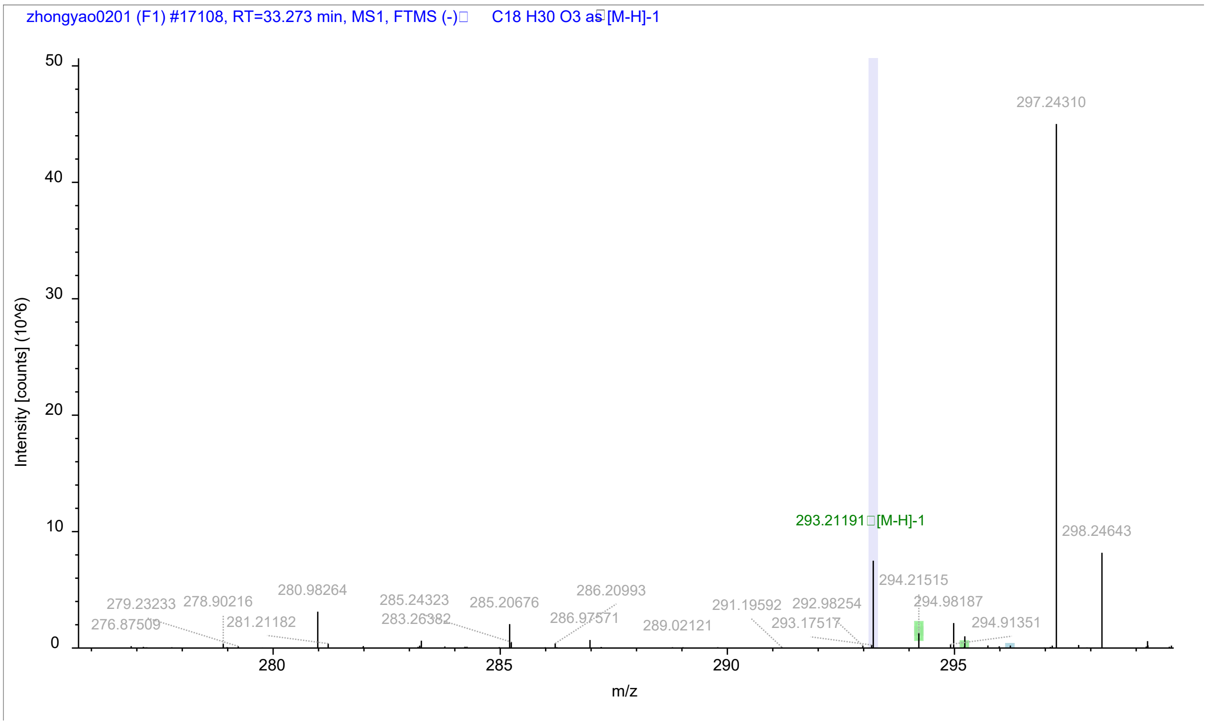

Figure S34. The MS^1 spectrum of 13-hydroxy-9*Z*,11*E*,15*Z*-octadecatrienoic acid (**17**)

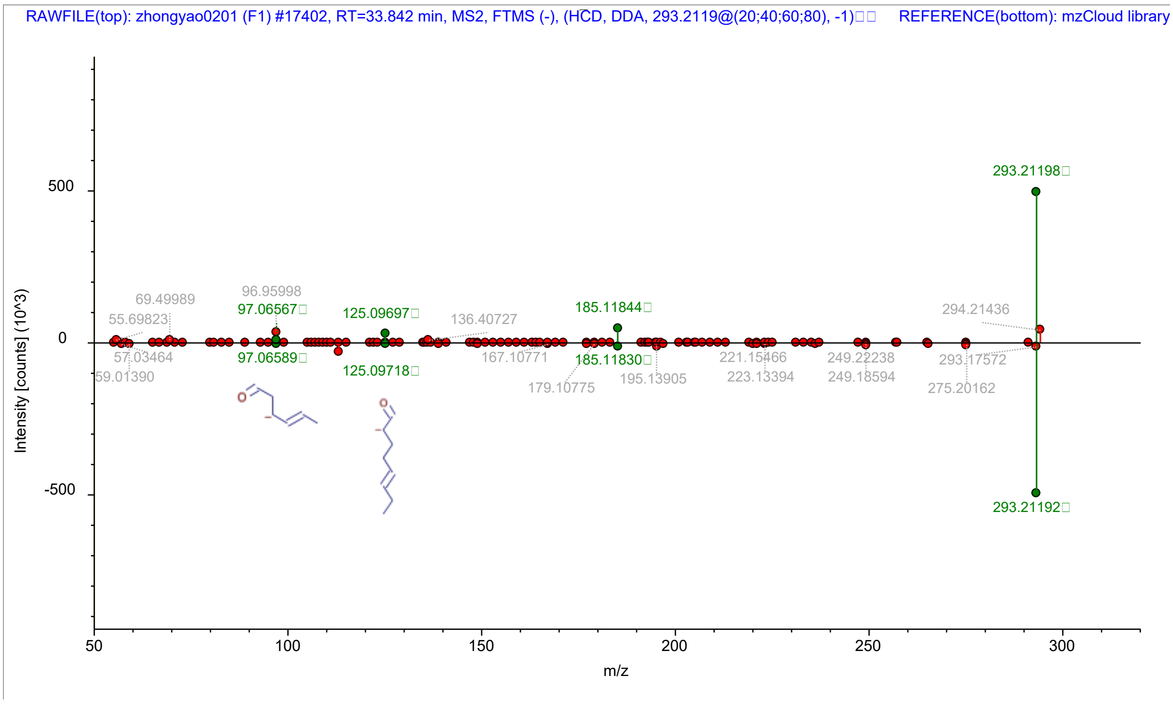

Figure S35. The MS[2] spectrum of 13-hydroxy-9*Z*,11*E*,15*Z*-octadecatrienoic acid (**17**)

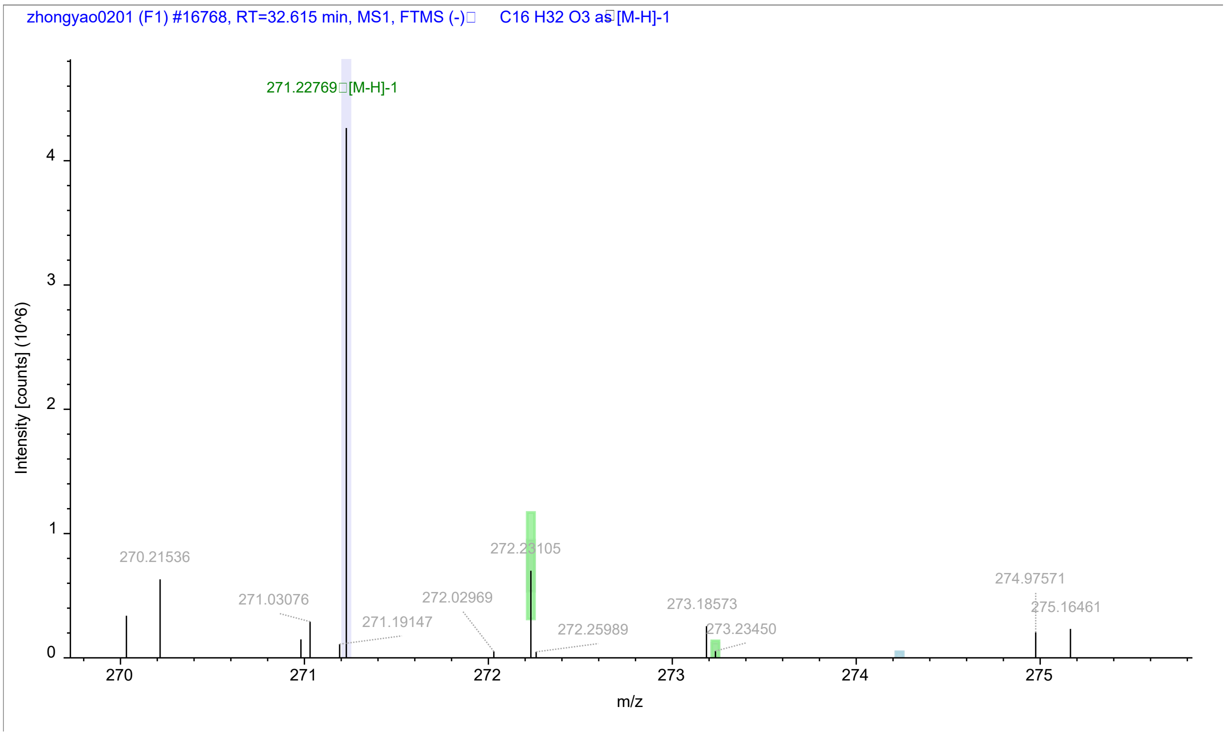

Figure S36. The MS[1] spectrum of 16-hydroxyhexadecanoic acid (**18**)

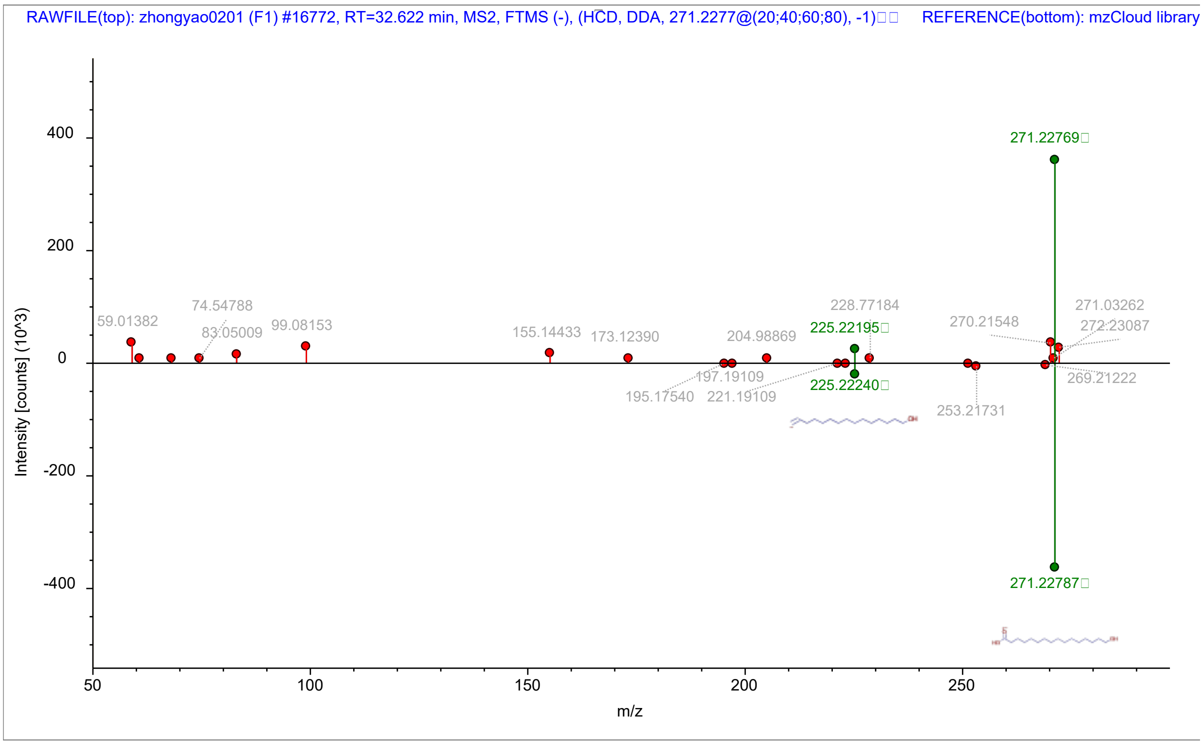

Figure S37. The MS^2 spectrum of 16-hydroxyhexadecanoic acid (**18**)

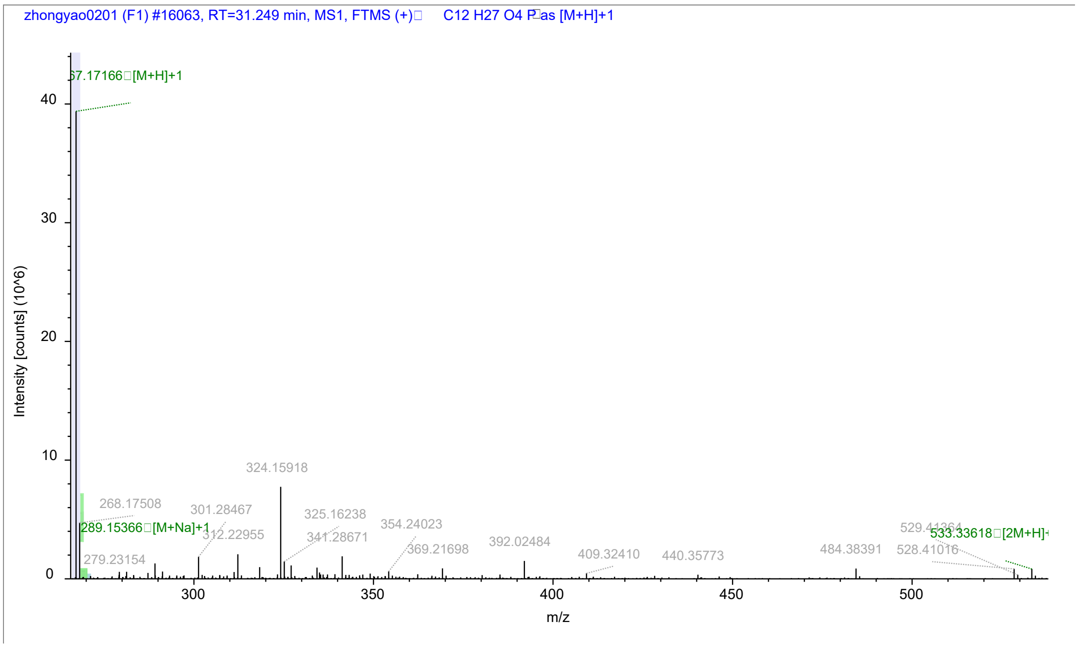

Figure S38. The MS^1 spectrum of tributyl phosphate (**19**)

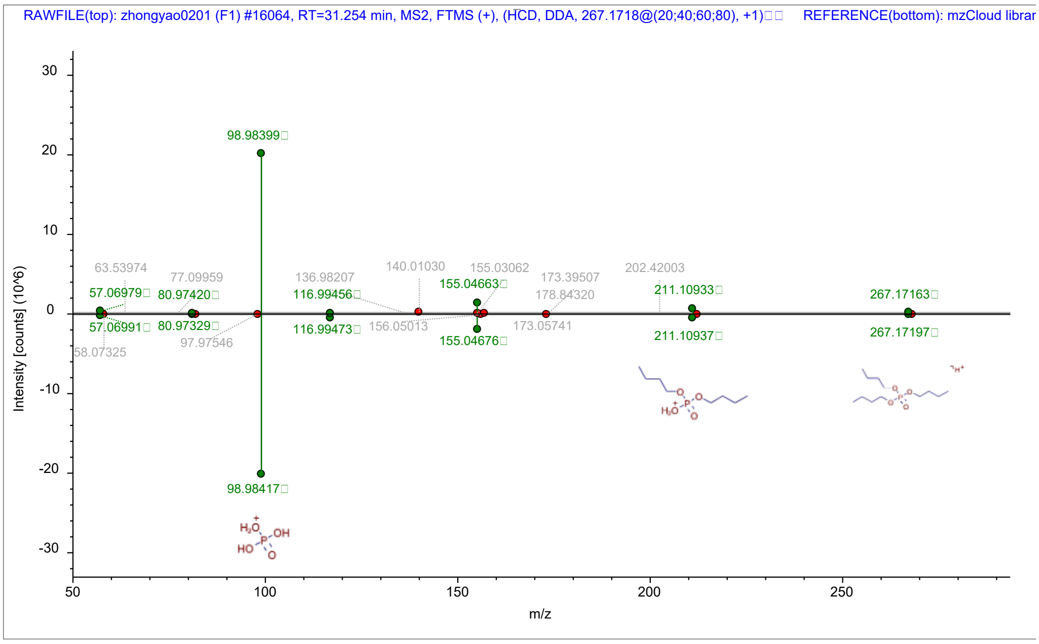

Figure S39. The MS^2 spectrum of tributyl phosphate (**19**)

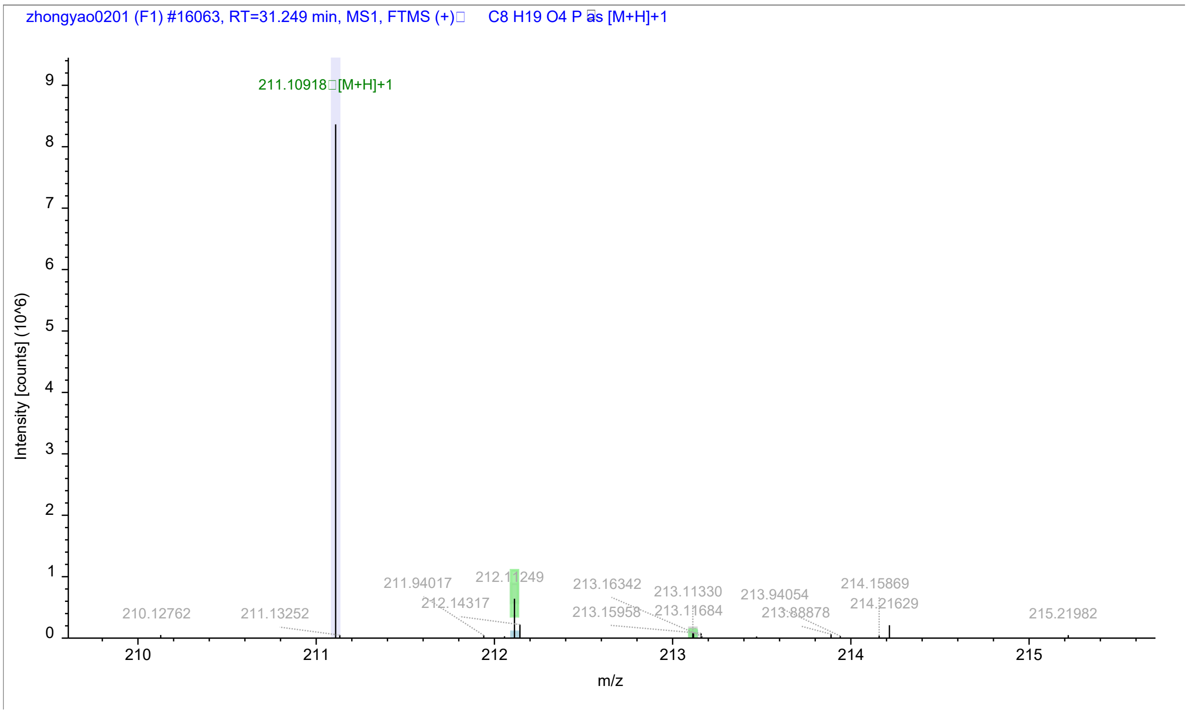

Figure S40. The MS^1 spectrum of dibutyl phosphate (**20**)

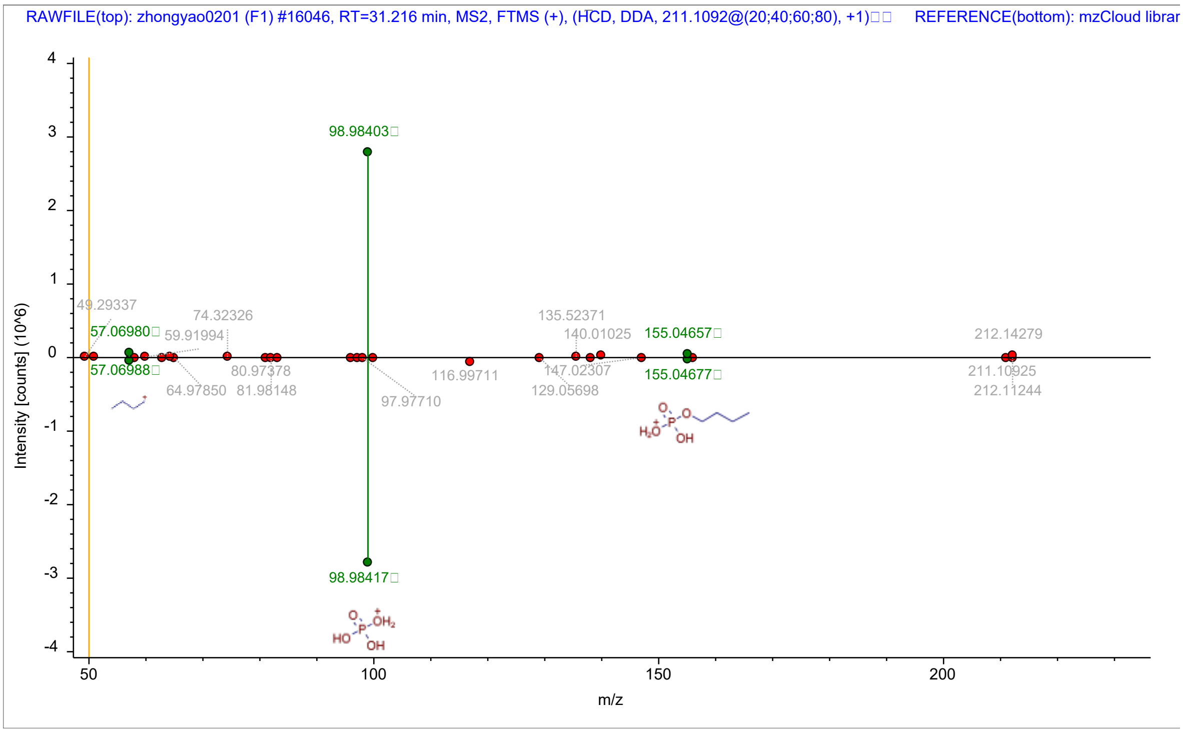

Figure S41. The MS^2 spectrum of dibutyl phosphate (**20**)

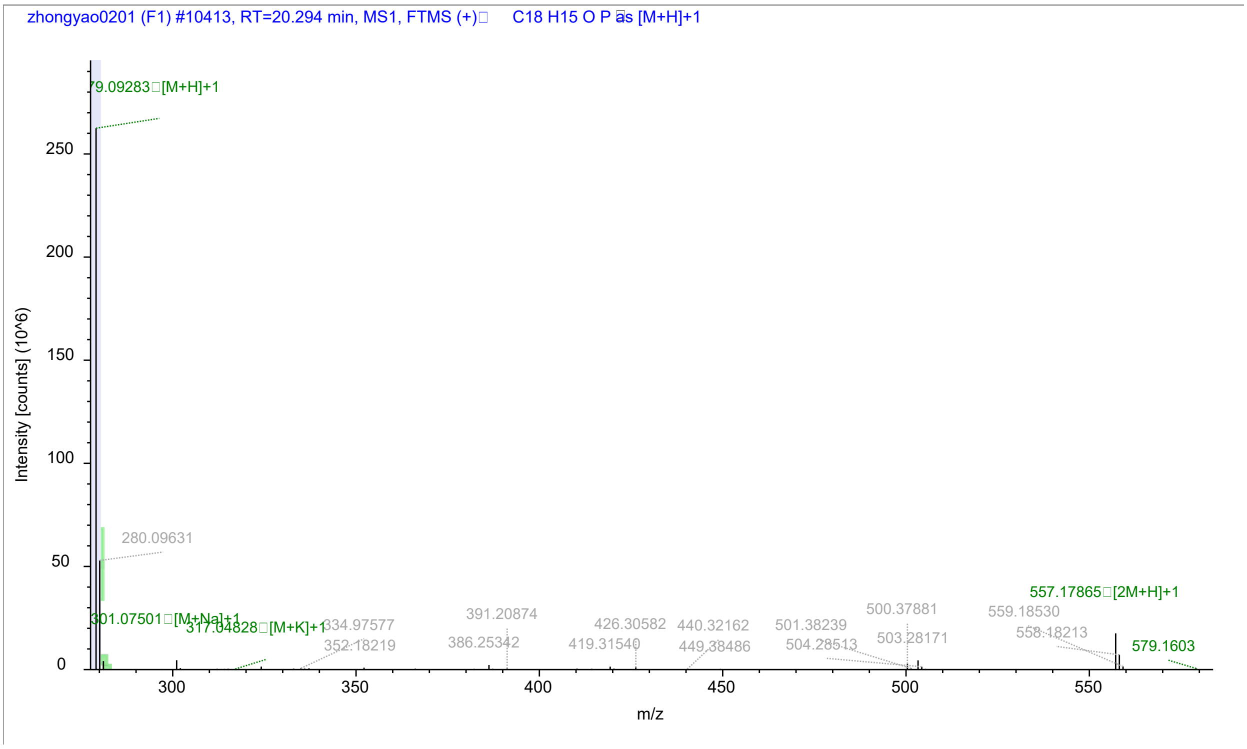

Figure S42. The MS^1 spectrum of triphenylphosphine oxide (**21**)

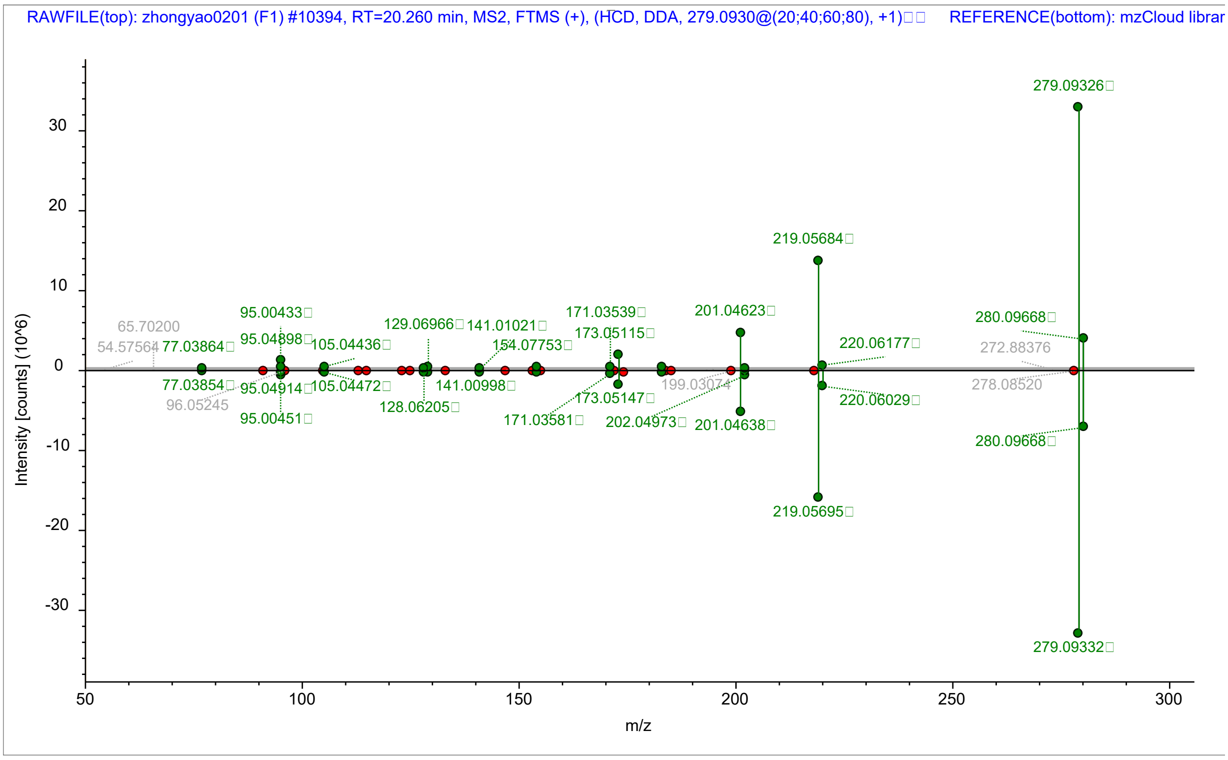

Figure S43. The MS^2 spectrum of triphenylphosphine oxide (**21**)

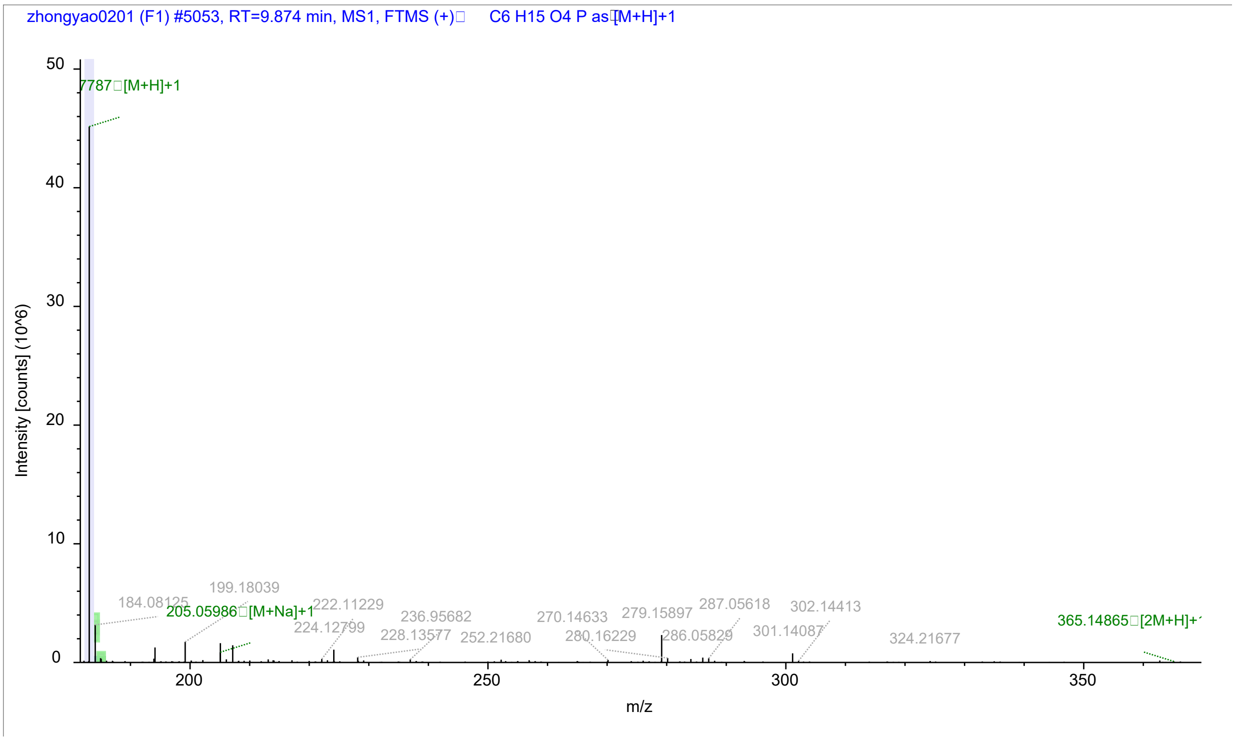

Figure S44. The MS^1 spectrum of triethyl phosphate (**22**)

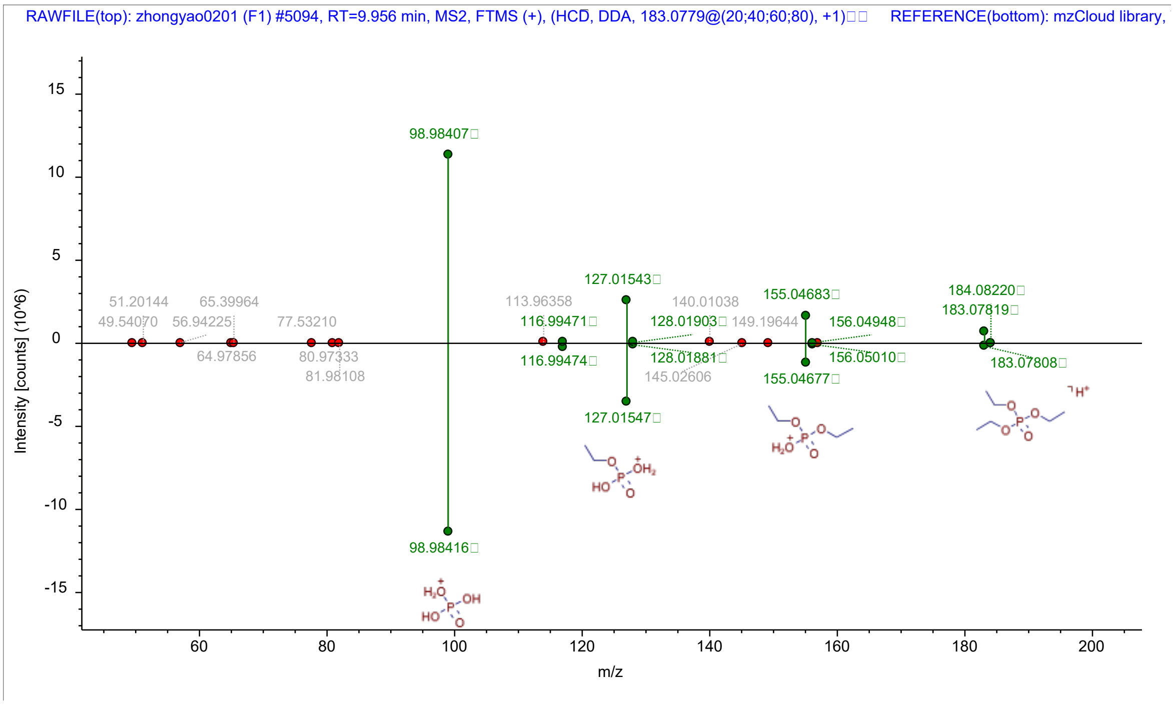

Figure S45. The MS^2 spectrum of triethyl phosphate (**22**)

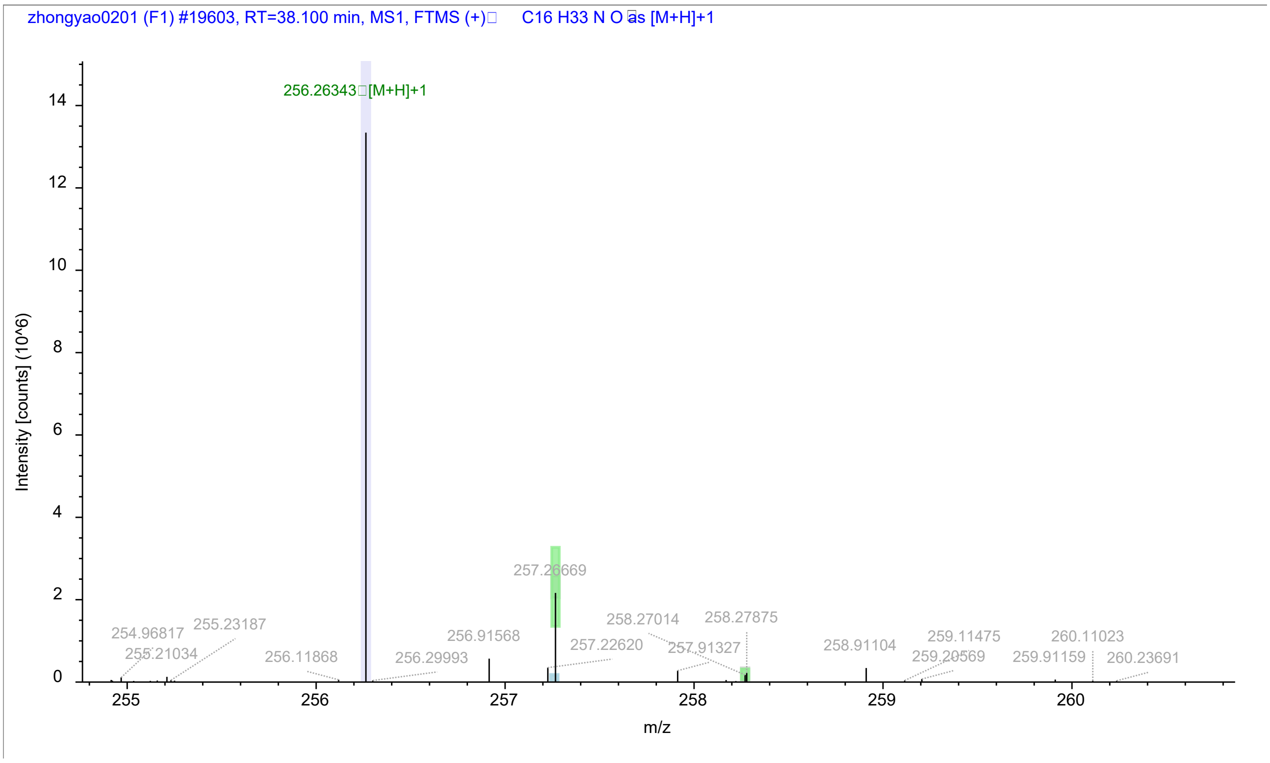

Figure S46. The MS^1 spectrum of hexadecanamide (**23**)

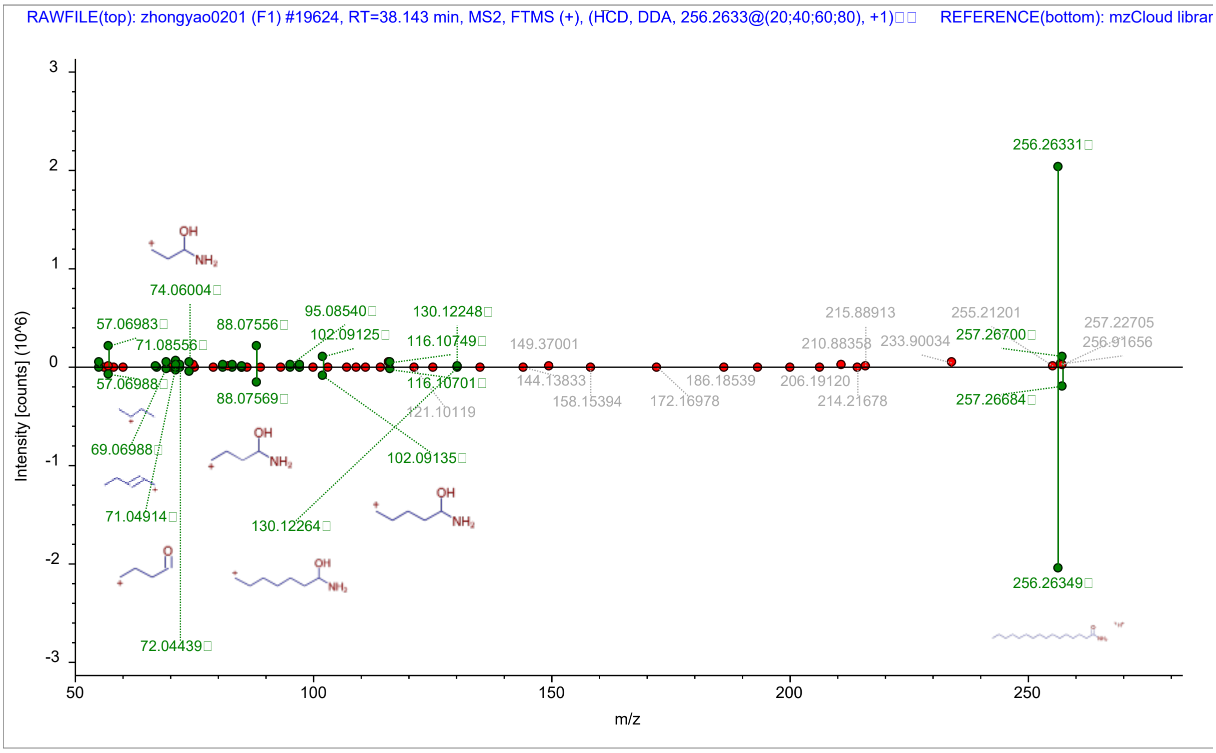

Figure S47. The MS^2 spectrum of hexadecanamide (**23**)

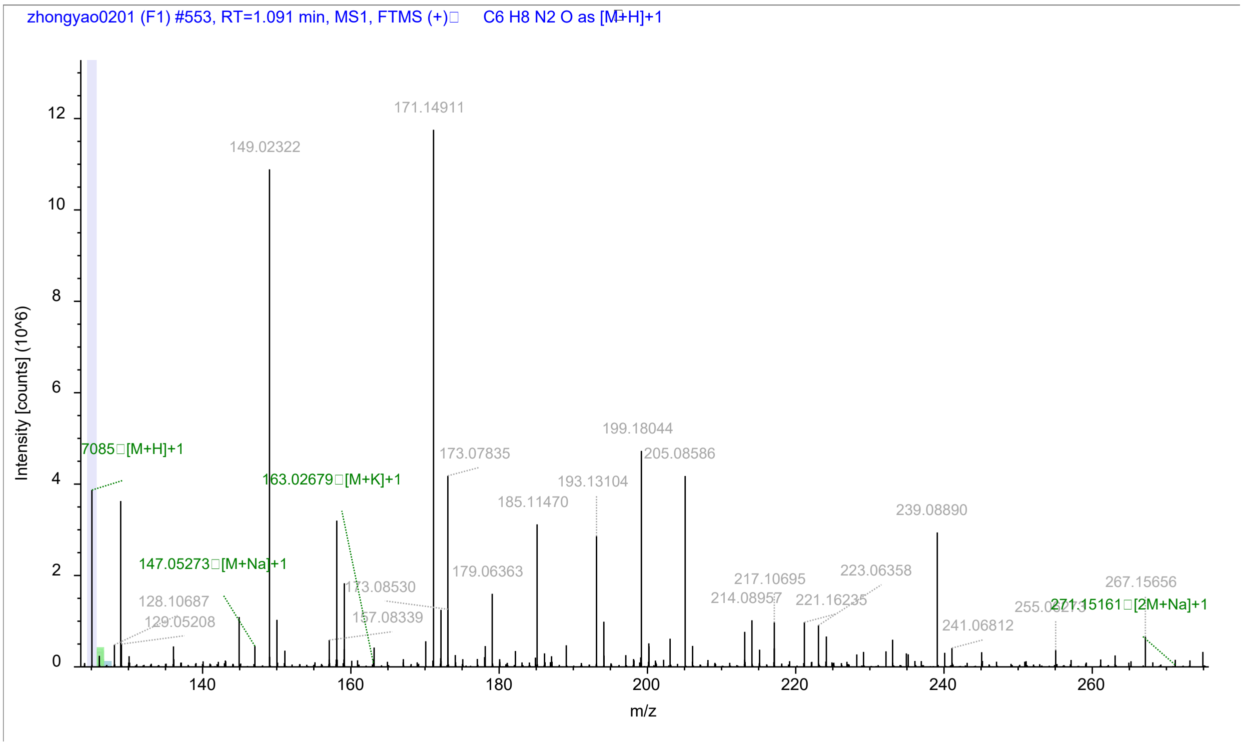

Figure S48. The MS^1 spectrum of 4,6-dimethyl-2-hydroxypyrimidine (**24**)

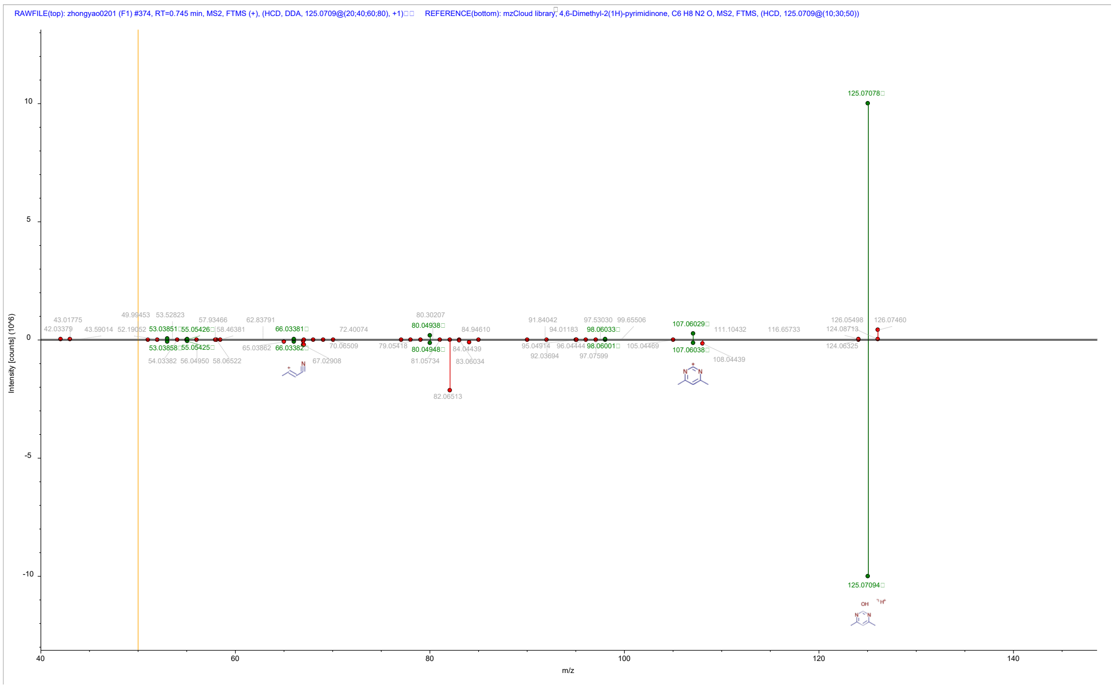

Figure S49. The MS^2 spectrum of 4,6-dimethyl-2-hydroxypyrimidine (**24**)

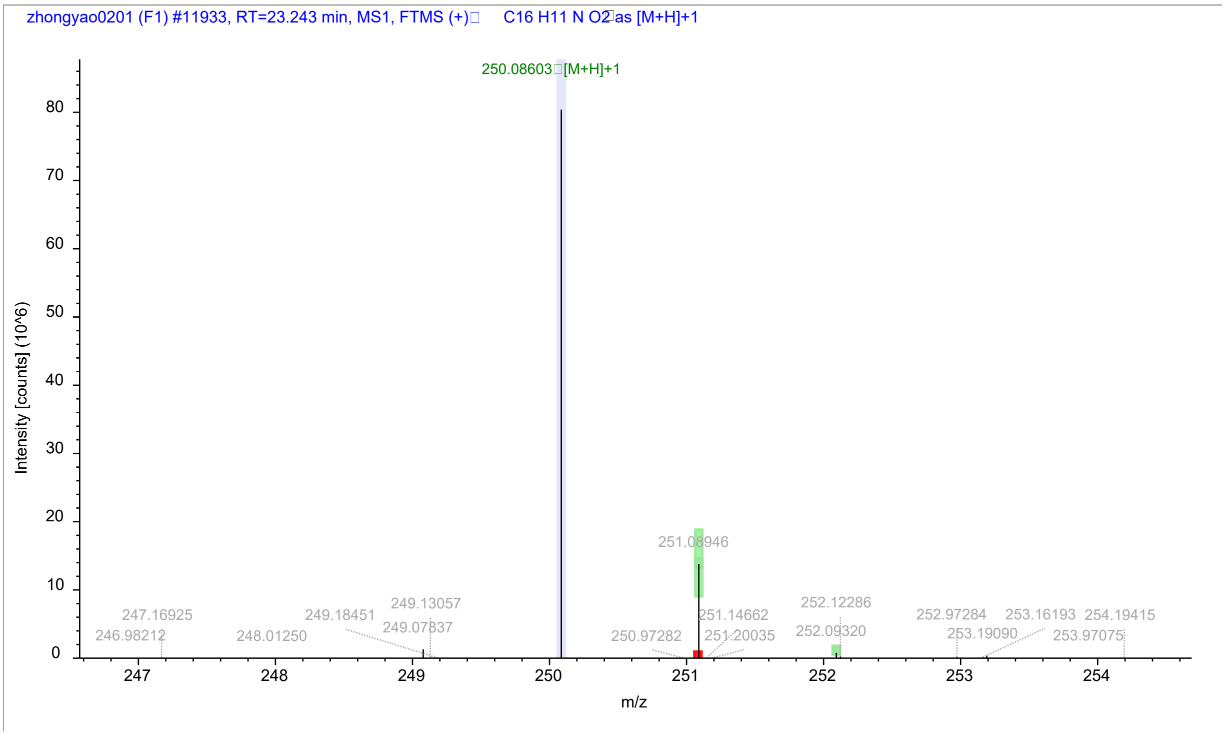

Figure S50. The MS^1 spectrum of cinchophen (**25**)

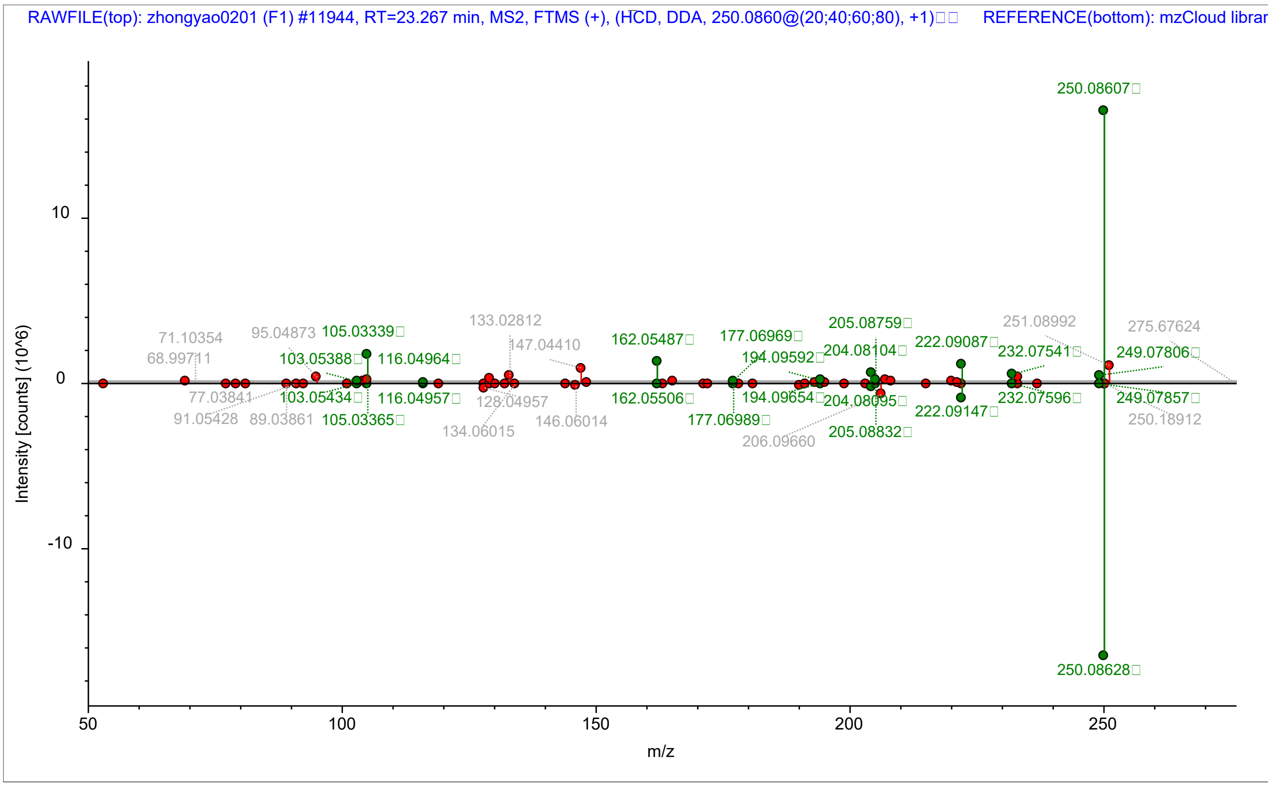

Figure S51. The MS^2 spectrum of cinchophen (**25**)

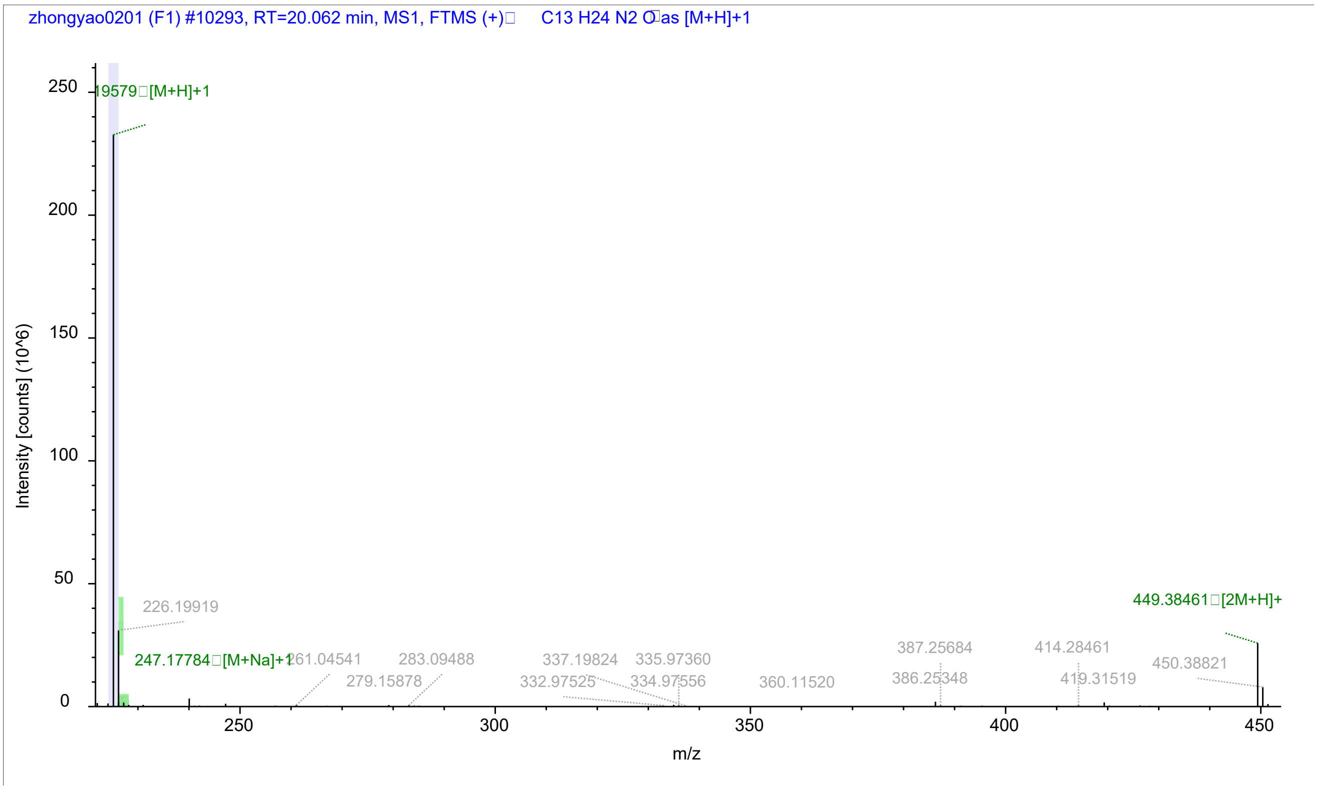

Figure S52. The MS^1 spectrum of N, N-dicyclohexylurea (**26**)

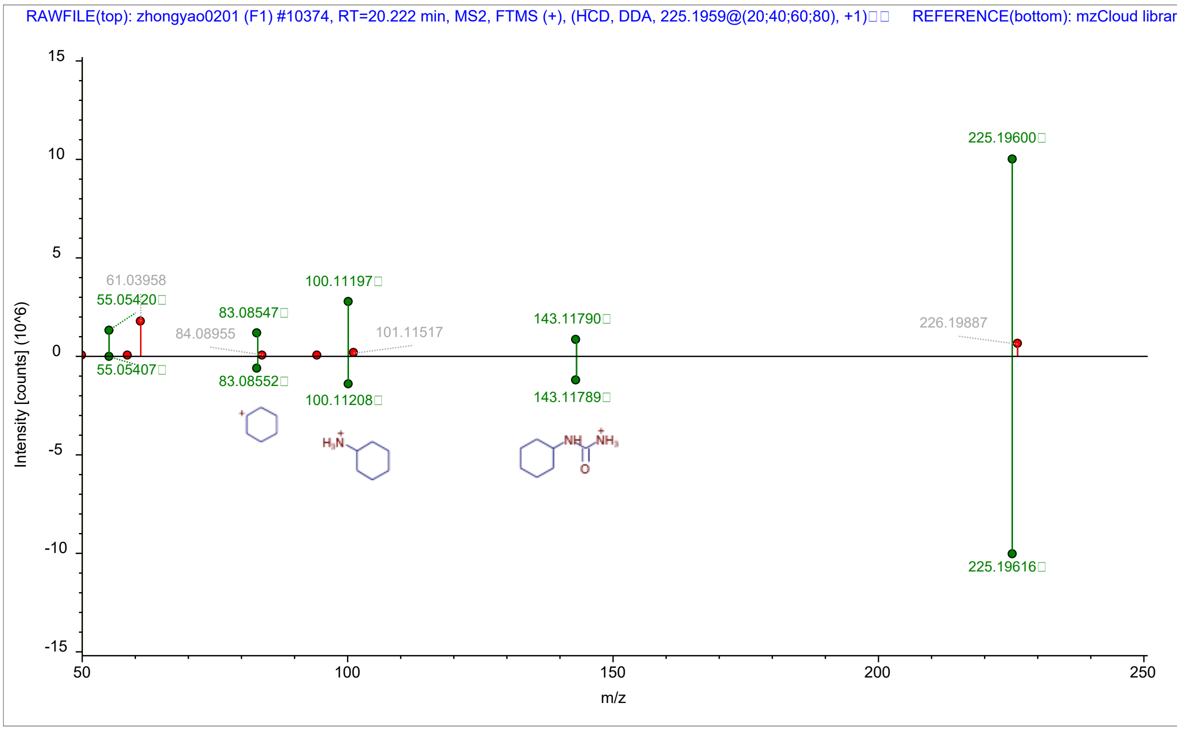

Figure S53. The MS^2 spectrum of N, N-dicyclohexylurea (**26**)

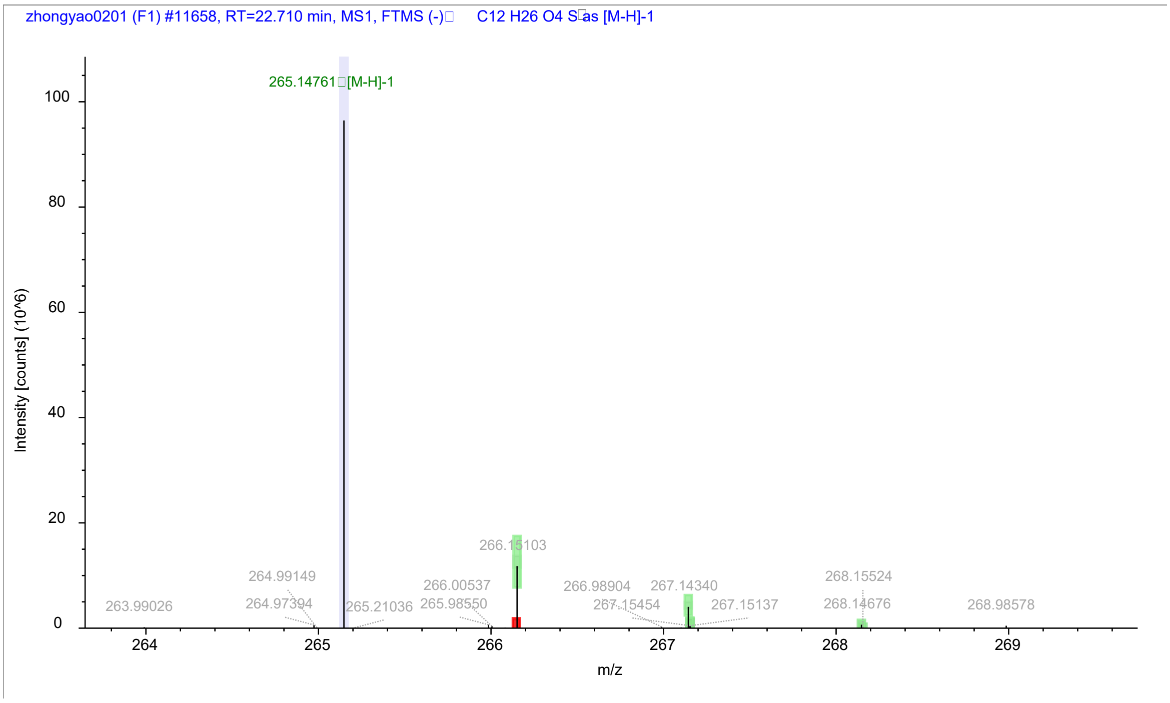

Figure S54. The MS^1 spectrum of dodecyl sulfate (**27**)

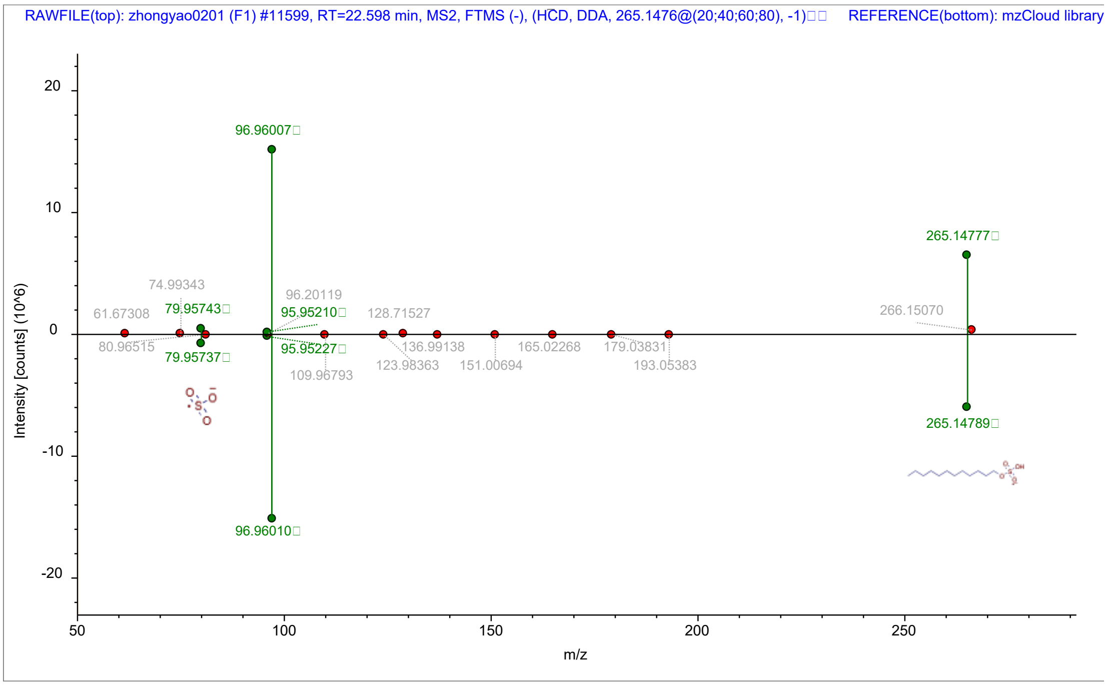

Figure S55. The MS^2 spectrum of dodecyl sulfate (**27**)

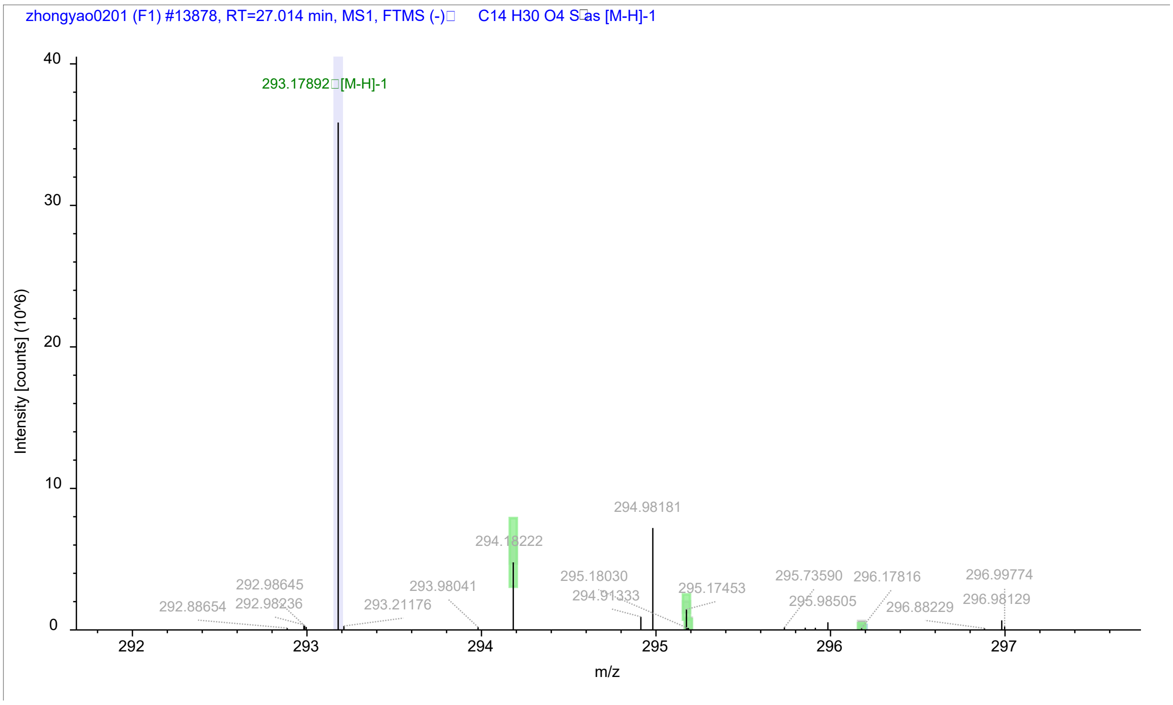

Figure S56. The MS^1 spectrum of myristyl sulfate (**28**)

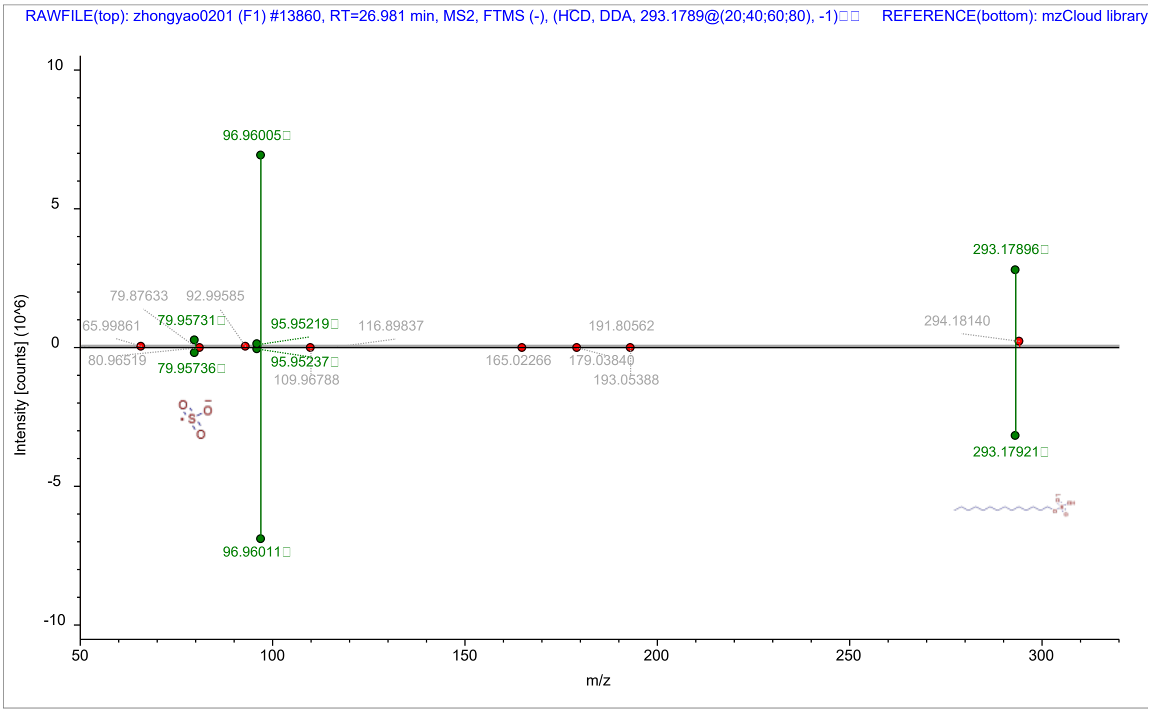

Figure S57. The MS^2 spectrum of myristyl sulfate (**28**)

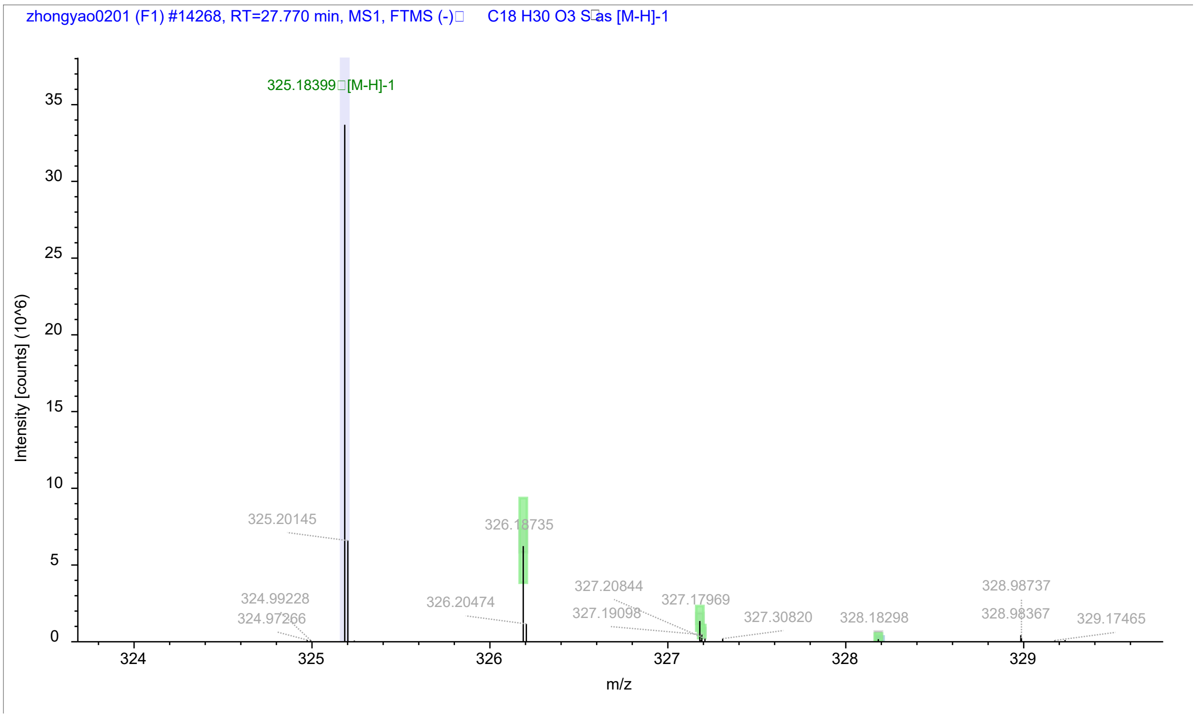

Figure S58. The MS^1 spectrum of 4-dodecylbenzenesulfonic acid (**29**)

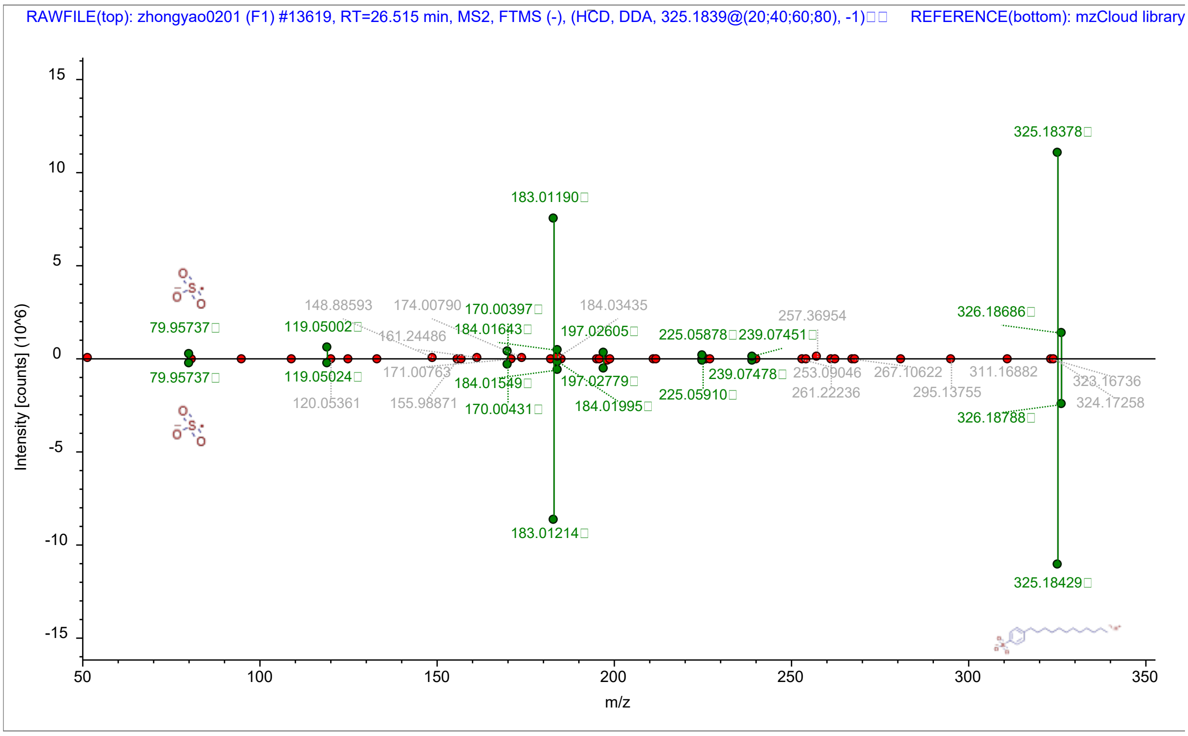

Figure S59. The MS^2 spectrum of 4-dodecylbenzenesulfonic acid (**29**)

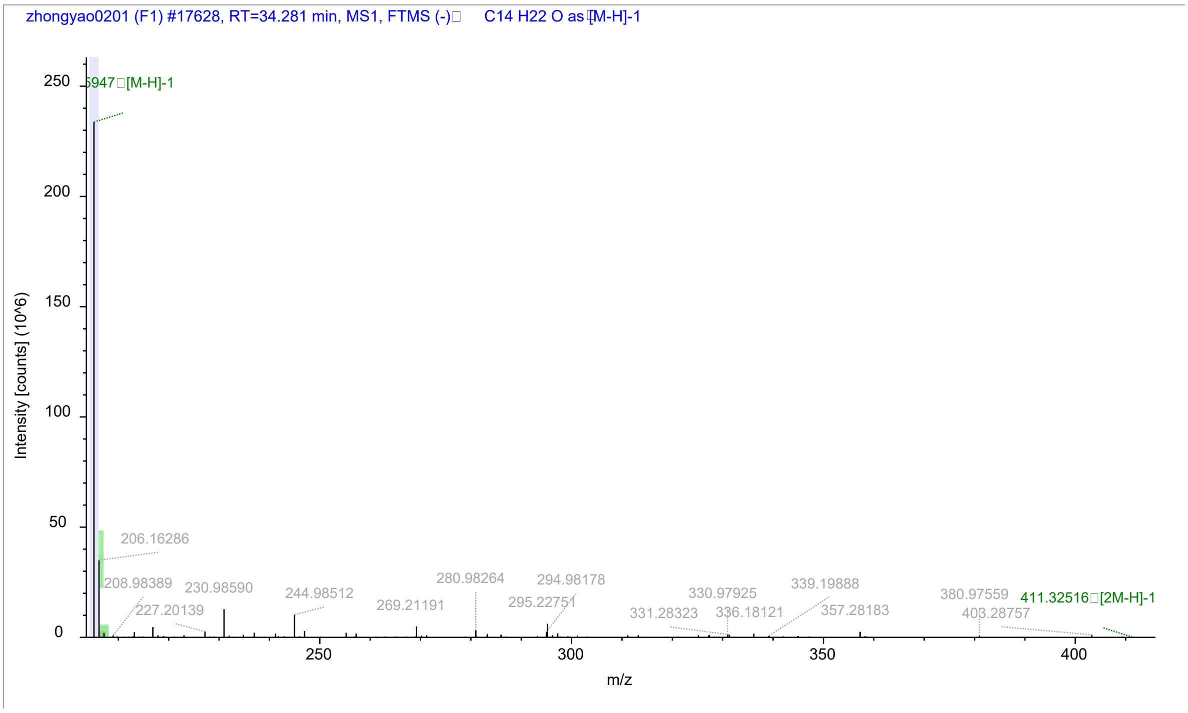

Figure S60. The MS^1 spectrum of 2,4-di-tert-butylphenol (**30**)

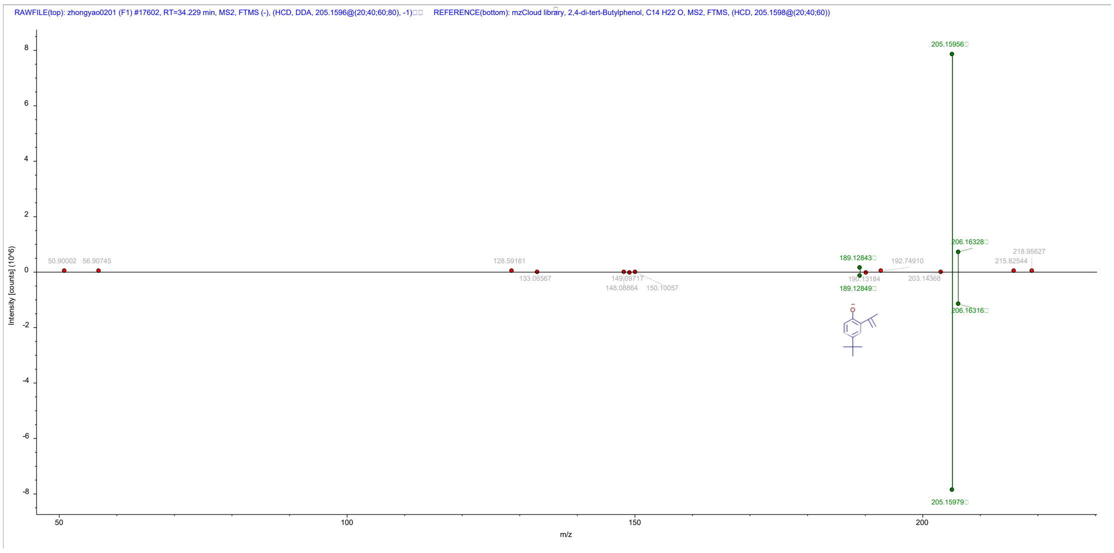

Figure S61. The MS^2 spectrum of 2,4-di-tert-butylphenol (**30**)

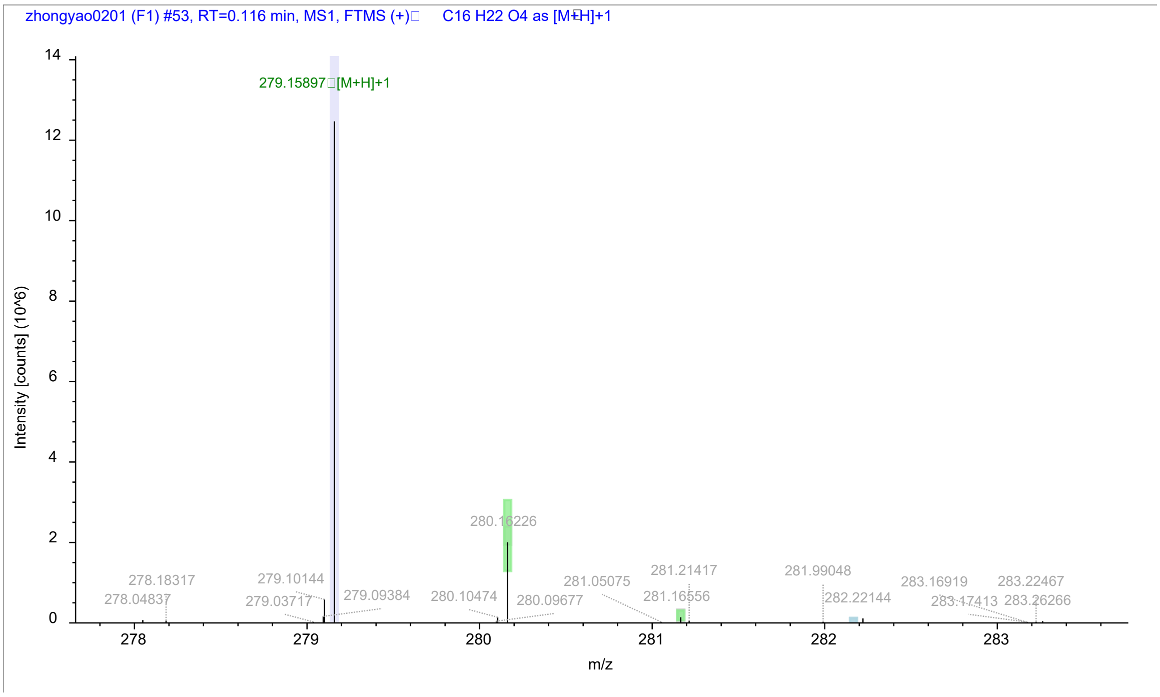

Figure S62. The MS^1 spectrum of dibutyl phthalate (**31**)

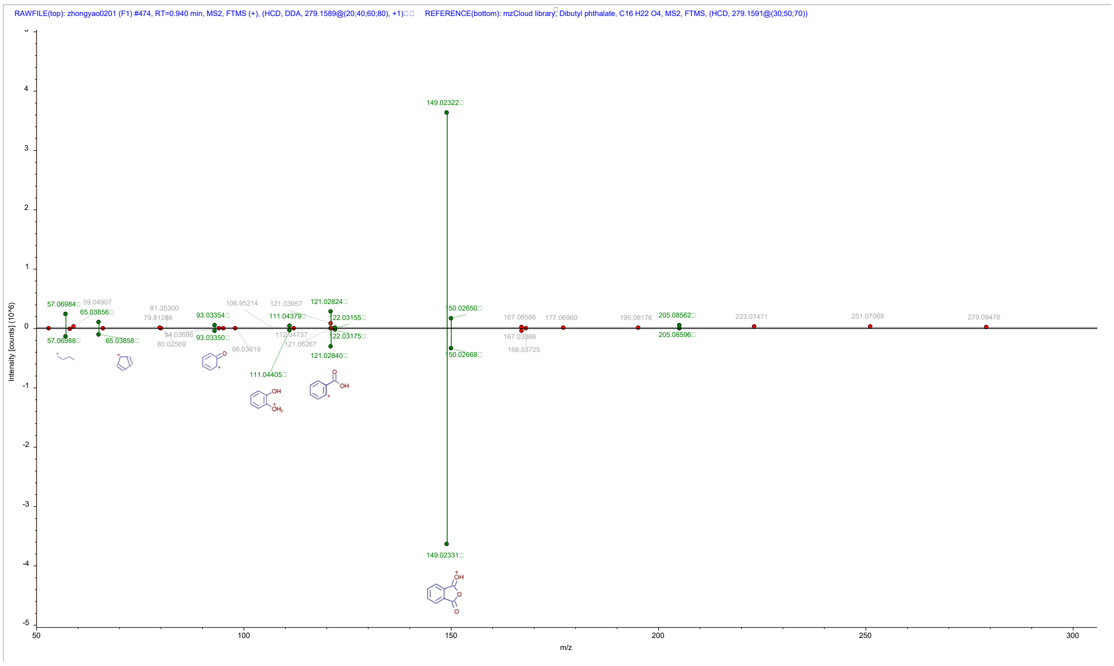

Figure S63. The MS^2 spectrum of dibutyl phthalate (**31**)

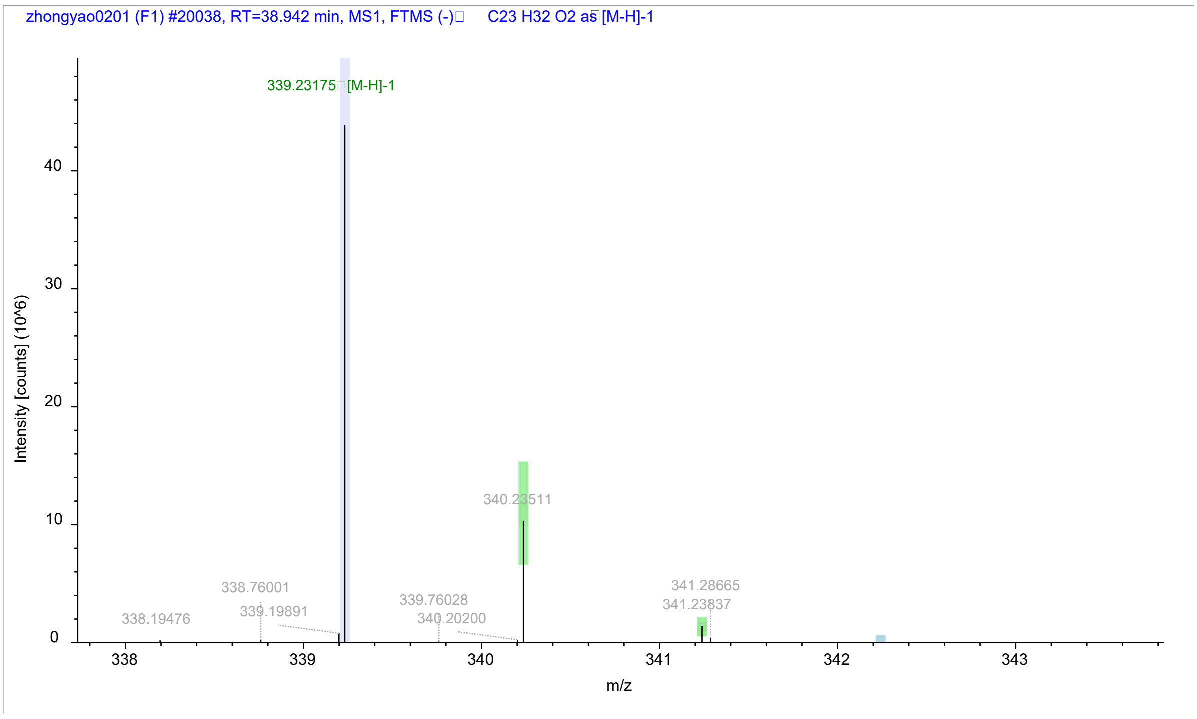

Figure S64. The MS^1 spectrum of 2,2'-methylenebis(4-methyl-6-tert-butylphenol) (**32**)

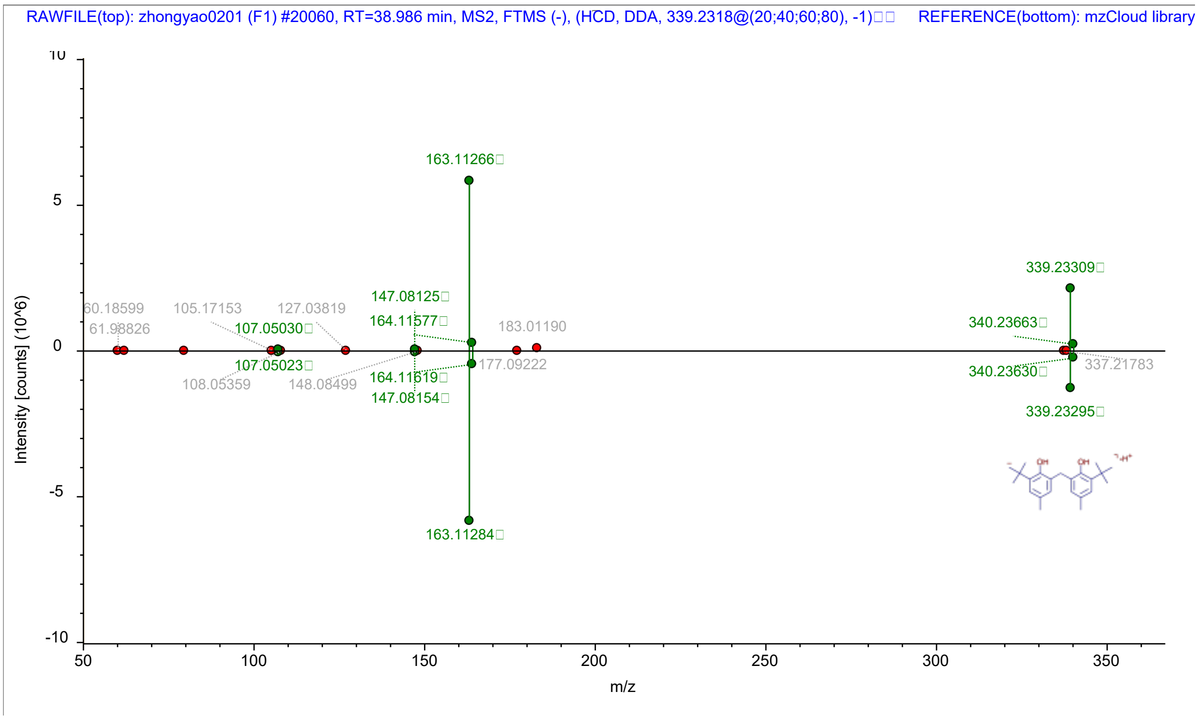

Figure S65. The MS[2] spectrum of 2,2'-methylenebis(4-methyl-6-tert-butylphenol) (**32**)

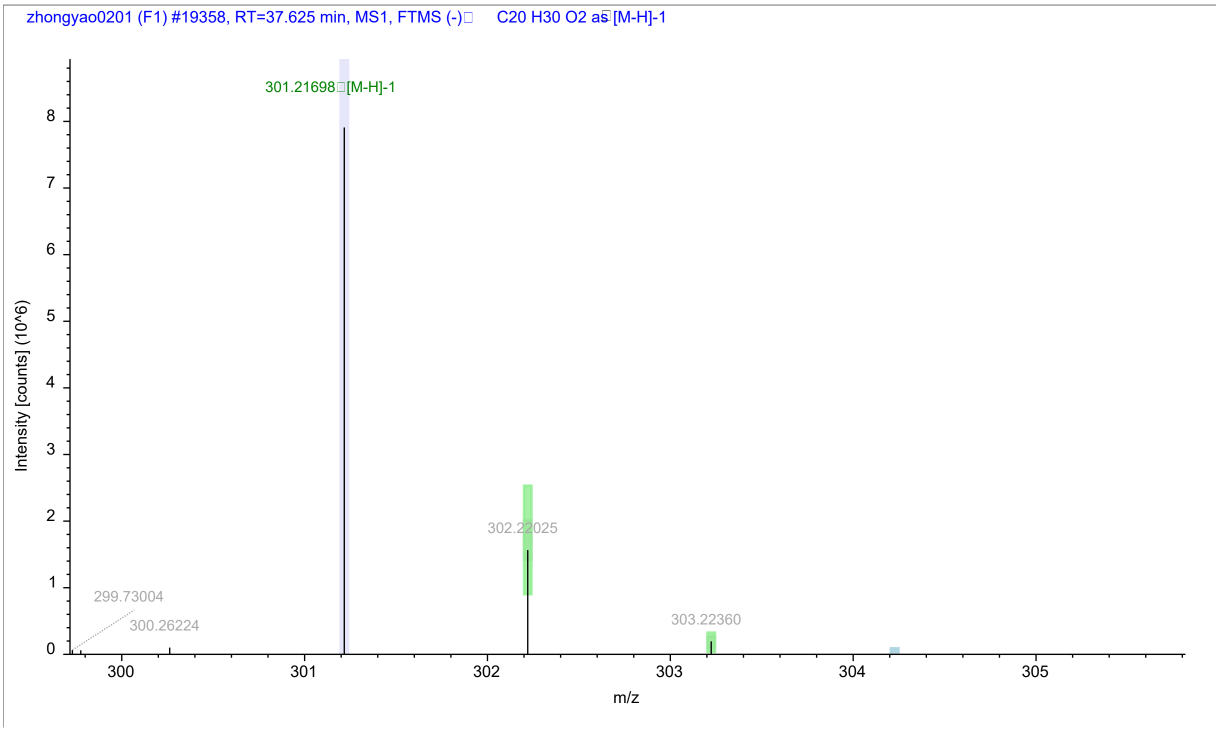

Figure S66. The MS[1] spectrum of abietic acid (**33**)

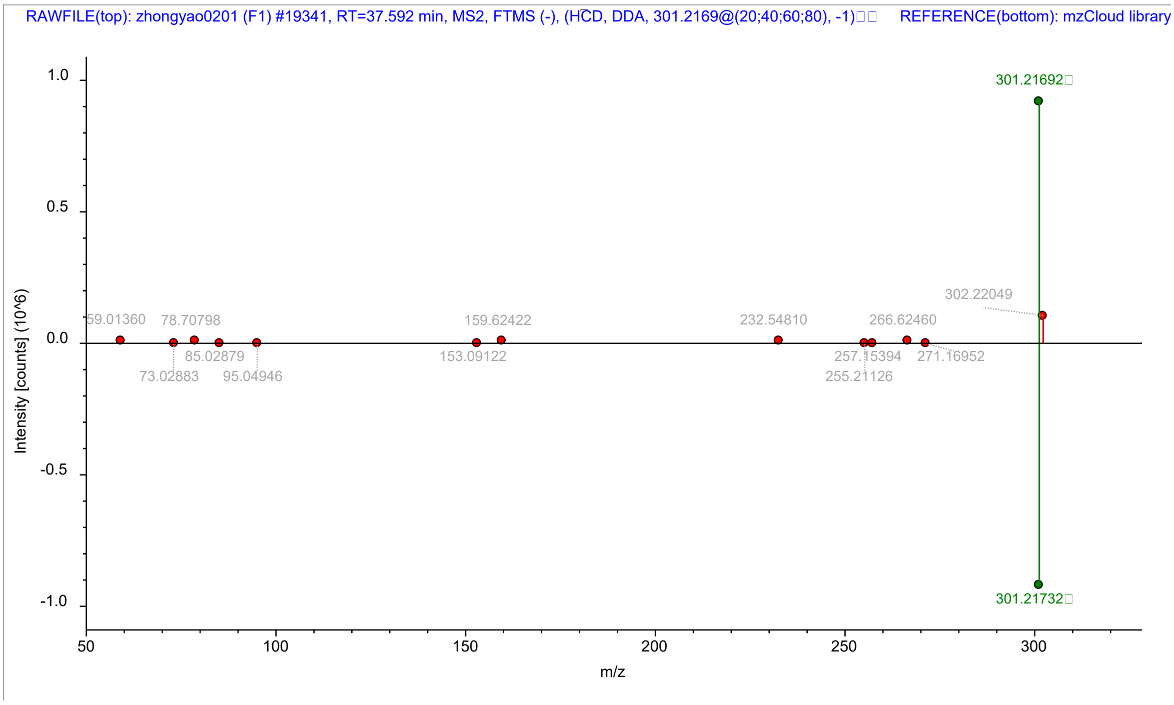

Figure S67. The MS^2 spectrum of abietic acid (**33**)

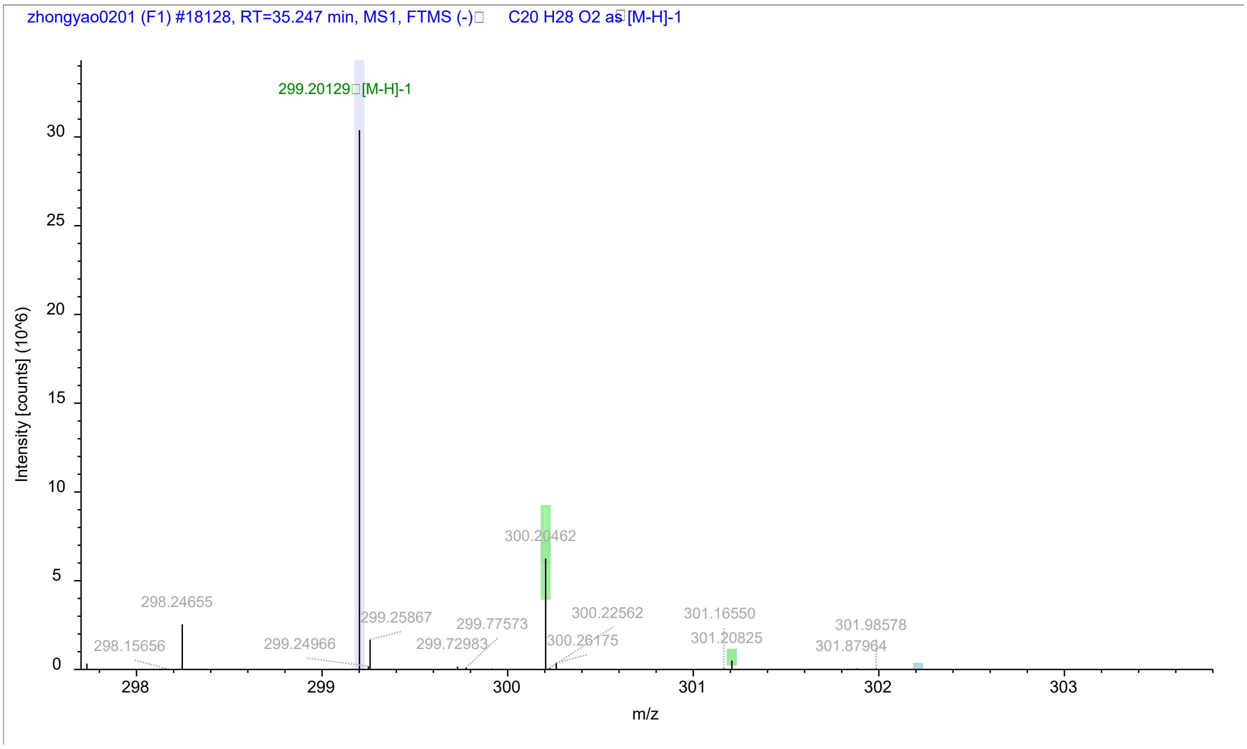

Figure S68. The MS^1 spectrum of tretinoin (**34**)

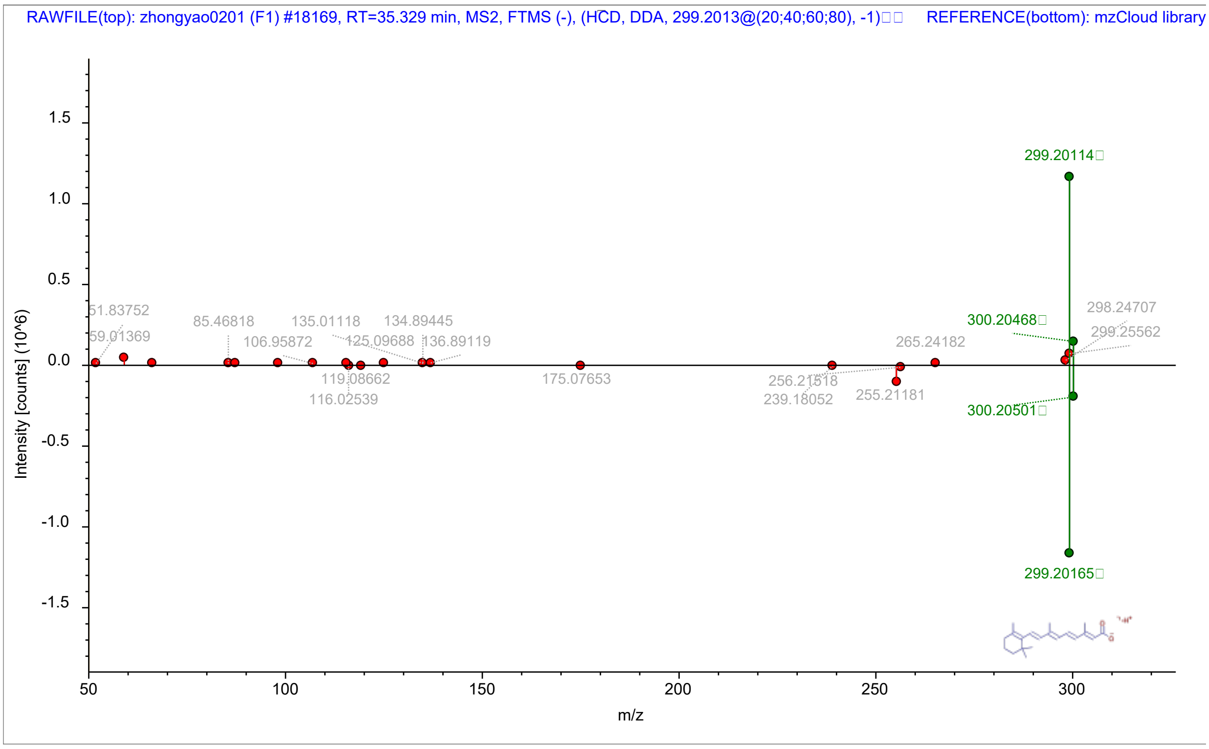

Figure S69. The MS^2 spectrum of tretinoin (**34**)

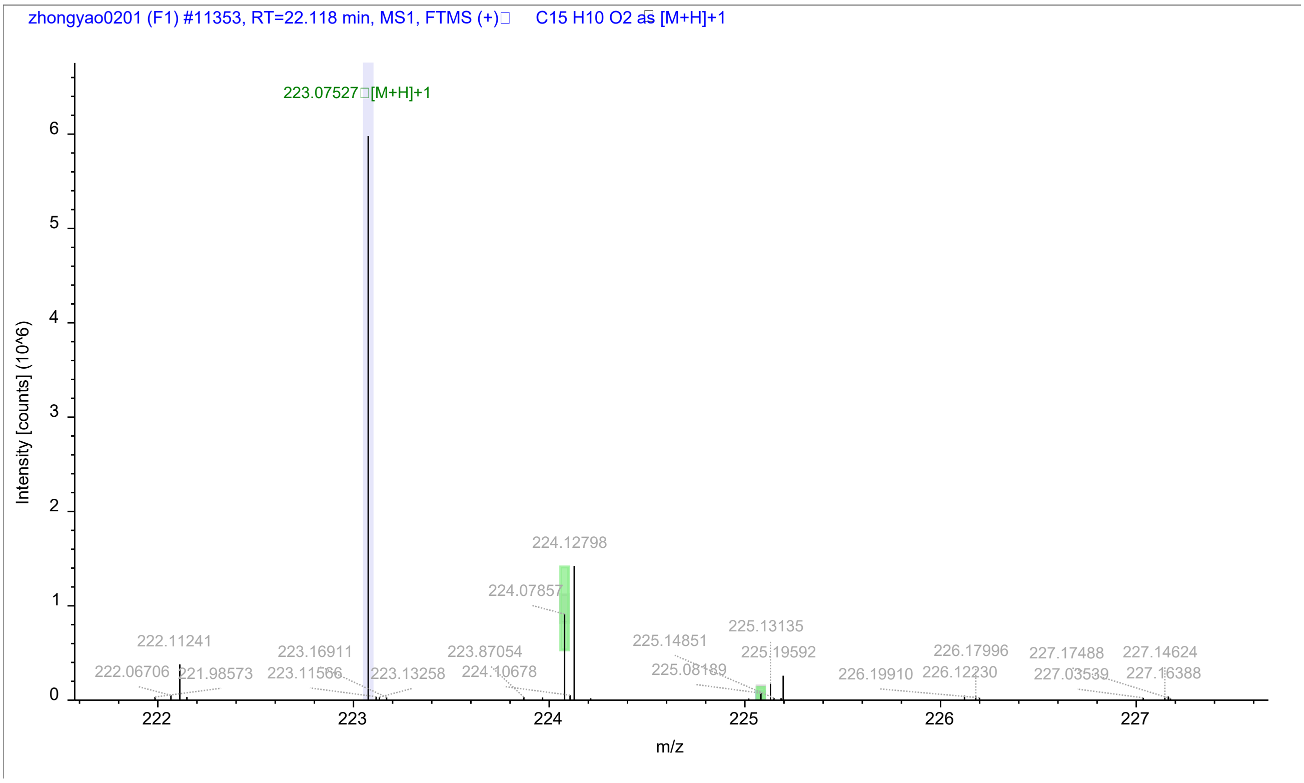

Figure S70. The MS^1 spectrum of flavone (**35**)

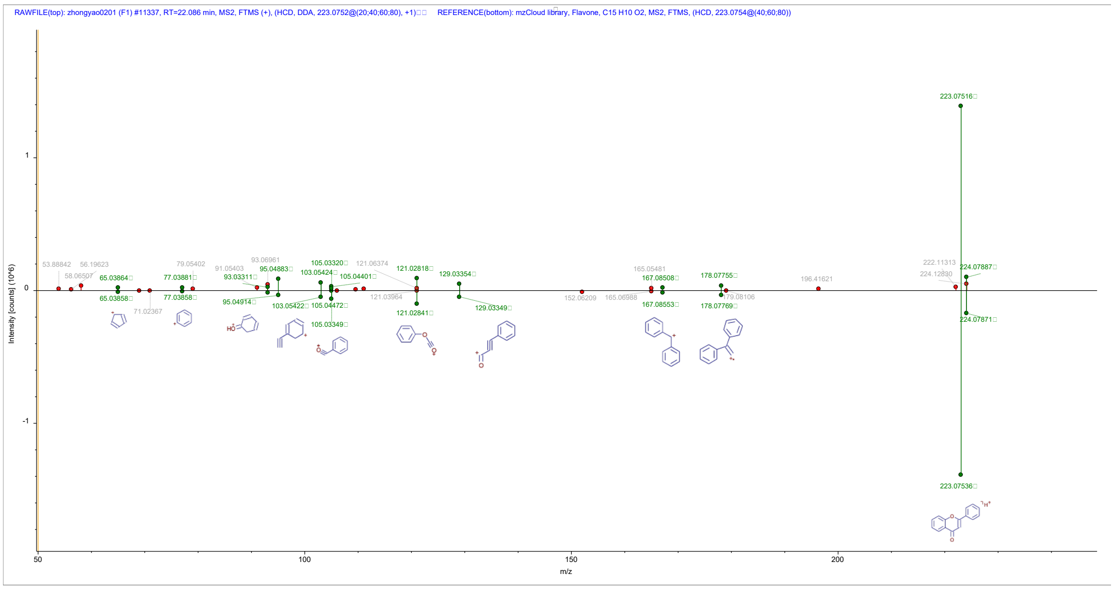

Figure S71. The MS^2 spectrum of flavone (**35**)

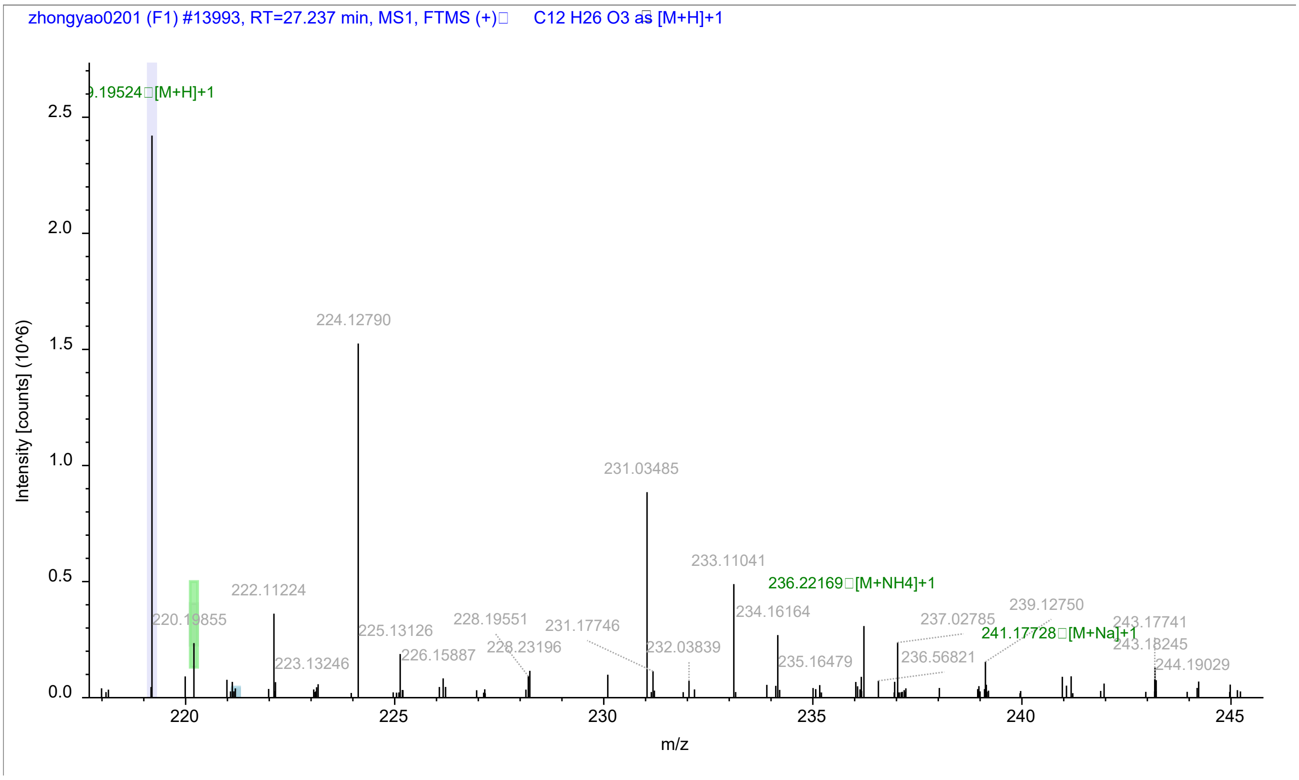

Figure S72. The MS^1 spectrum of bis(2-butoxyethyl) ethe (**36**)

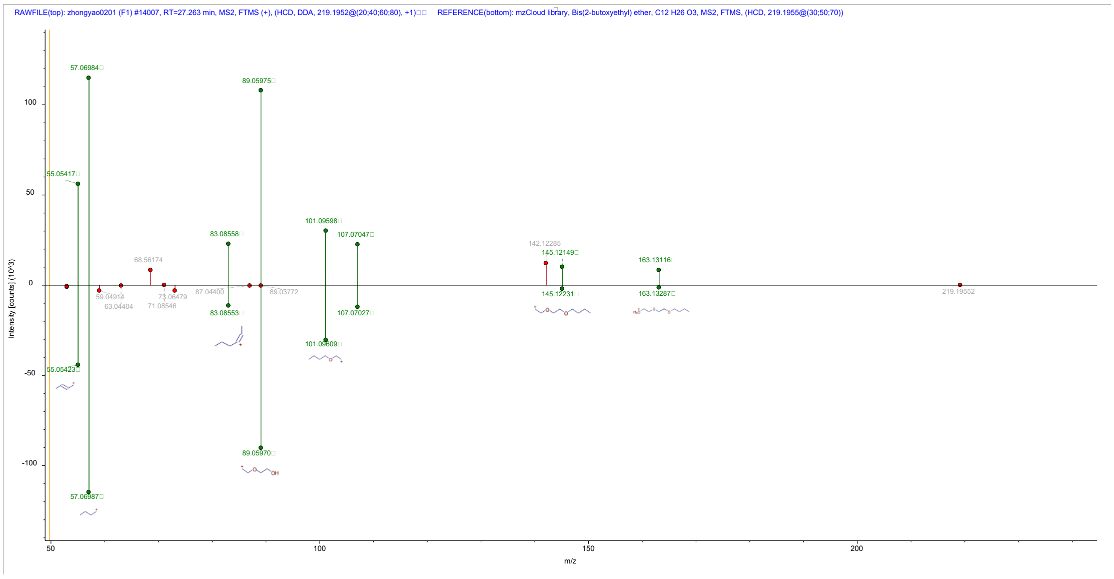

Figure S73. The MS^2 spectrum of bis(2-butoxyethyl) ethe (**36**)

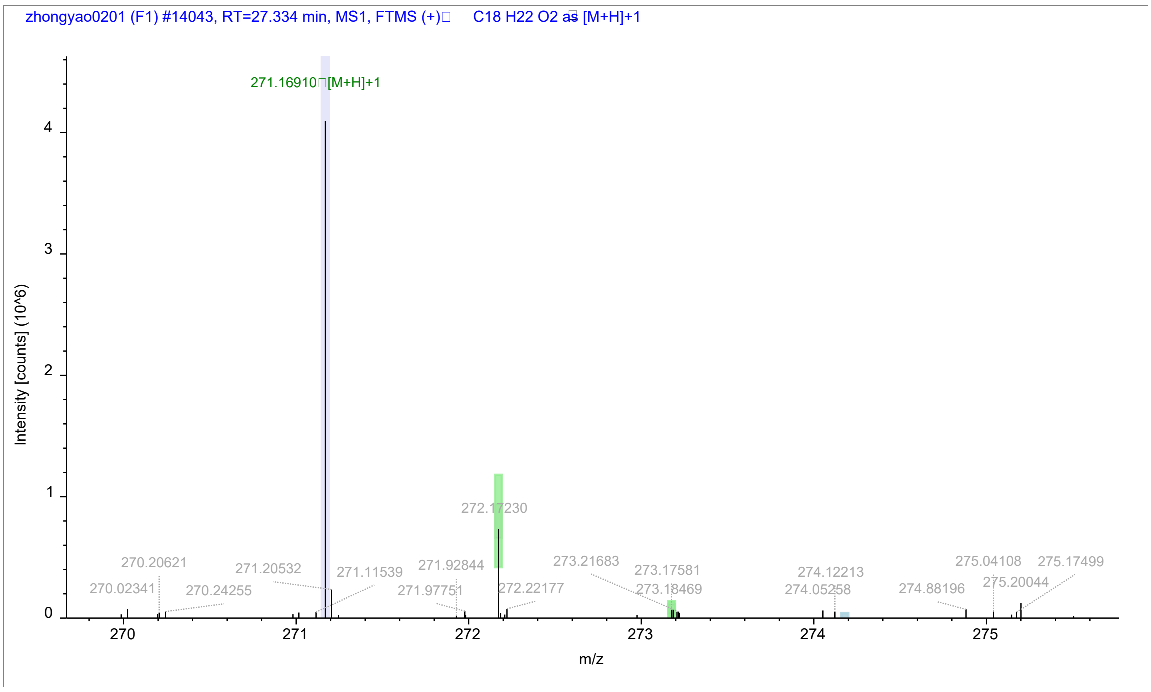

Figure S74. The MS^1 spectrum of trenbolone (**37**)

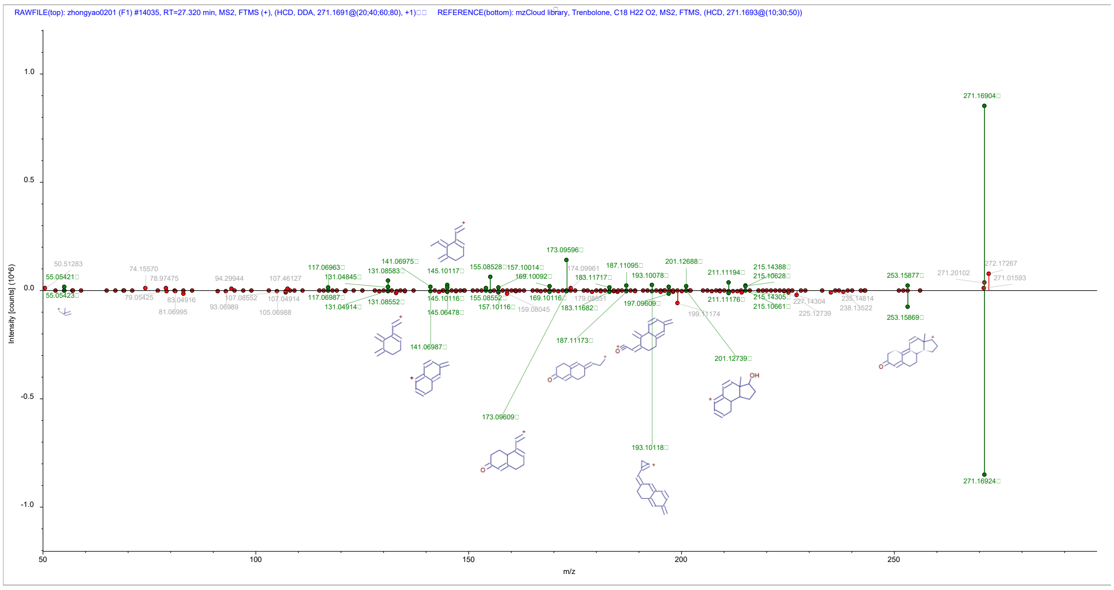

Figure S75. The MS^2 spectrum of trenbolone (**37**)

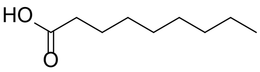
1. Nonanoic acid

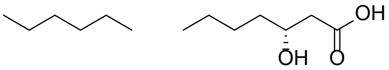
2. 3-Hydroxy myristic acid

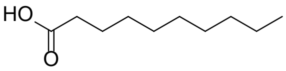
3. Decanoic acid

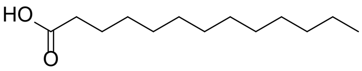
4. Tridecylic acid

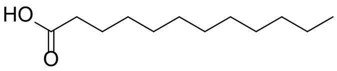
5. Lauric acid

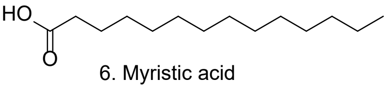
6. Myristic acid

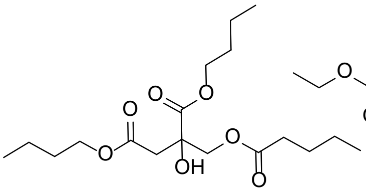
7. Tributyl citrate

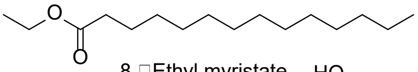
8. Ethyl myristate

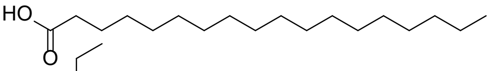
9. Stearic acid

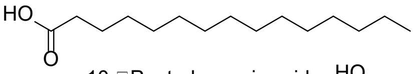
10. Pentadecanoic acid

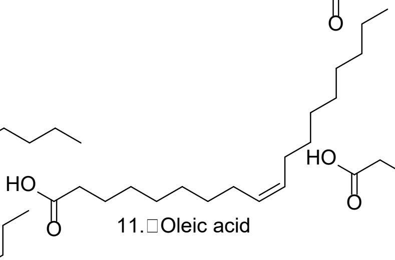
11. Oleic acid

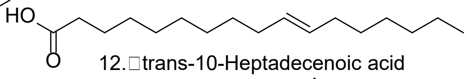
12. trans-10-Heptadecenoic acid

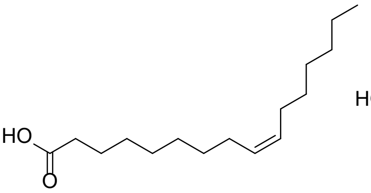
13. Palmitoleic acid

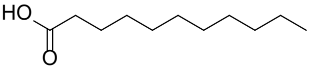
14. Undecanoic acid

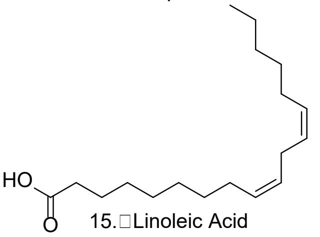
15. Linoleic Acid

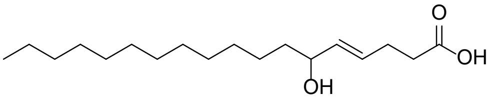
16. (E)-6-hydroxyoctadec-4-enoic acid

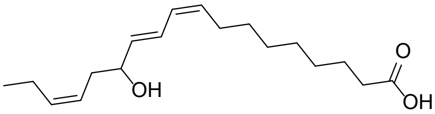
17. 13-hydroxy-9Z,11E,15Z-octadecatrienoic acid

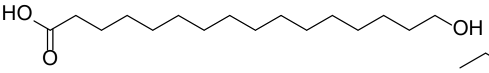
18. 16-Hydroxyhexadecanoic acid

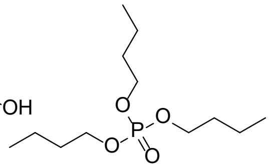
19. Tributyl phosphate

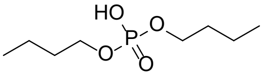
20. Dibutyl phosphate

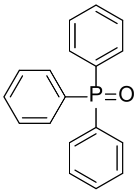

21. Triphenylphosphine oxide

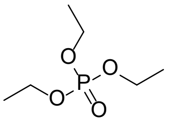

22. Triethyl phosphate

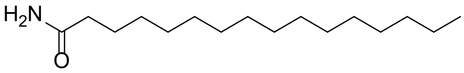

23. Hexadecanamide

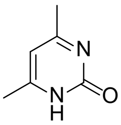

24. 4,6-Dimethyl-2-hydroxypyrimidine

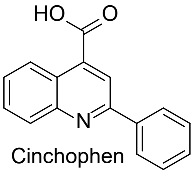

25. Cinchophen

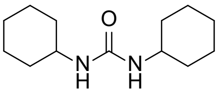

26. N,N-Dicyclohexylurea

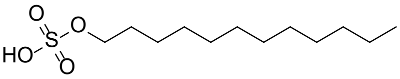

27. Dodecyl sulfate

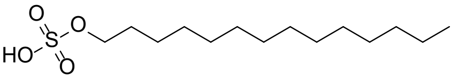

28. Myristyl sulfate

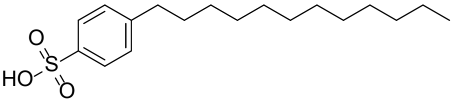

29. 4-Dodecylbenzenesulfonic acid

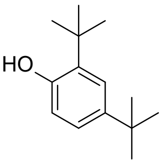

30. 2,4-Di-tert-butylphenol

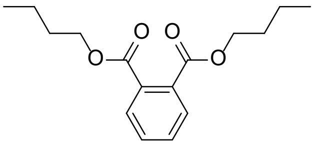

31. Dibutyl phthalate

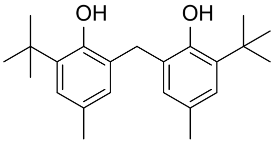

32. 2,2'-Methylenebis (4-methyl-6-tert-butylphenol)

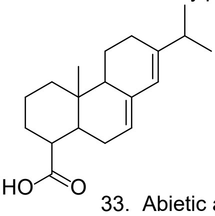

33. Abietic acid

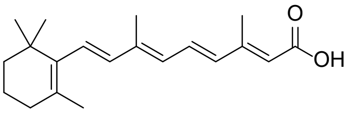

34. Tretinoin

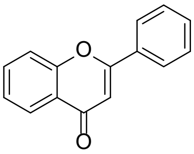

35. Flavone

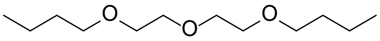

36. Bis(2-butoxyethyl) ether

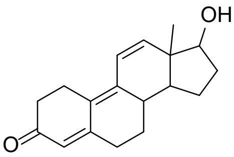

37. Trenbolone

Figure S76. The chemical structures of compounds (**1-37**)
